# Untargeted Metabolomics Reveals Major Patterns of Metabolic Shifts in Potato Seed Tubers during Storage

**DOI:** 10.1007/s11540-026-10083-2

**Published:** 2026-06-05

**Authors:** Chunmei Zou, Ric C. H. de Vos, Roland Mumm, Henriëtte D. L. M. van Eekelen, Aurin M. Vos, Alejandro Thérèse Navarro, Robert D. Hall, Martin K. van Ittersum, Willemien J. M. Lommen, Paul C. Struik

**Affiliations:** 1 Centre for Crop Systems Analysis, Wageningen University & Research, P.O. Box 430, 6700 AK Wageningen, The Netherlands; 2 Plant Production Systems, Wageningen University & Research, P.O. Box 430, 6700 AK Wageningen, The Netherlands; 3 Bioscience, Wageningen University & Research, P.O. Box 16, 6700 AA Wageningen, The Netherlands; 4 Plant Breeding, Wageningen University & Research, P.O. Box 386, 6700 AJ Wageningen, The Netherlands; 5 Present Address: Rijk Zwaan, 4793 RS Rijnaart, The Netherlands

**Keywords:** Cultivar, GC–MS, LC–MS, Physiological age, Storage, *Solanum tuberosum*

## Abstract

To obtain comprehensive insight into metabolic changes in potato seed tubers during storage and to identify patterns associated with physiological ageing, we analysed tuber metabolite composition using untargeted metabolomics based on gas and liquid chromatography-mass spectrometry (GC–MS and LC–MS). During two seasons, seed tubers of four contrasting cultivars (Agria, Festien, Innovator, and Lady Claire) were produced in a single field and after harvest, stored at different temperatures. During storage, the number of detected secondary metabolites increased progressively, particularly at higher temperatures (7–17 ˚C). Principal component analysis (PCA) revealed clear cultivar-specific metabolic profiles, with the starch cultivar Festien distinctly separated from the consumption cultivars, primarily caused by different amino acid composition. PCA further highlighted the impact of cold storage (4 ˚C) on primary metabolism, especially the accumulation of reducing sugars, as well as the combined effects of storage duration and elevated temperature on secondary metabolites, notably glycoalkaloids. Factor analysis (FA) supported these findings, with most metabolites strongly associated with the factor distinguishing cv. Festien from the other cultivars. Additionally, the leading factors captured cultivar-specific patterns and diverse trajectories reflecting the effects of storage duration and temperature. Together, these results provide a comprehensive overview of metabolic dynamics during storage and contribute to understanding the functional roles of metabolites in tuber physiological ageing.

**Supplementary Information:**

The online version contains supplementary material available at 10.1007/s11540-026-10083-2.

## Introduction

The potato (*Solanum tuberosum* L.) crop is usually propagated vegetatively, with seed tubers serving as the primary starting material for the next planting cycle. In most potato-growing regions, these seed tubers need to be stored for extended periods. Potato seed tubers are metabolically active, even during cold storage, progressing through several physiological phases including dormancy, apical dominance, multiple sprouting, and senescence (Struik and Wiersema 1999). This physiological ageing process is influenced not only by chronological age but also by external factors such as growth history and storage conditions (e.g., Struik 2018), with cultivar-specific effects (e.g., Zou et al. 2024). The physiological age of seed tubers strongly influences their vigour and is critical to subsequent crop performance and yield (Iritani et al. 1983; van der Zaag and van Loon 1987). Understanding the underlying metabolic processes is therefore vital to the potato production system.

Several metabolites have been reported to change during the physiological ageing of tubers, including increases in sucrose, malic acid, polyamines, and amino acids such as valine, lysine, and isoleucine, alongside general decreases in citric acid and abscisic acid (ABA) (Rumpf 1972; Shekhar and Iritani 1979; Apelbaum 1984; Reust and Aerny 1985; Suttle 1995; Biemelt et al. 2000; Fukuda et al. 2019). During tuber sprouting, elevated levels of polyamines (putrescine, spermidine, spermine), organic acids (citrate and succinate), and tuberonic acid have been observed, together with increased concentrations of phytohormones such as gibberellic acid, cytokinin, and a concurrent decline in ABA (Kaur-Sawhney et al. 1982; Suttle 1995, 2004; Viola et al. 2007; Suttle et al. 2011). Sprouting is also associated with substantial accumulation of glycoalkaloids, primarily α-solanine and α-chaconine (Friedman and Dao 1992). In addition, cold storage (e.g., below 7 ˚C) promotes the conversion of starch into reducing sugars, including glucose and fructose, a process known as cold-induced sweetening (reviewed by Sowokinos 2001). Despite these well-documented metabolic changes, no single metabolite or class of metabolites has been identified as a regulator of physiological ageing. This underscores the need for comprehensive approaches that capture broad metabolite profiles and account for their changes under multiple interacting factors influencing the ageing process.

Metabolomics technologies—particularly untargeted approaches that analyse all detected compounds—have become invaluable for studying plant metabolite composition and exploring metabolic networks (Hall 2006; Toubiana et al. 2013). Mass spectrometry (MS), coupled with gas chromatography (GC–MS) or liquid chromatography (LC–MS), has been increasingly used in metabolomics studies. GC–MS, typically applied following derivatisation of aqueous methanol extracts, enables the detection of a broad range of primary metabolites, including sugars, sugar alcohols, organic acids, amino acids, phosphorylated intermediates, and certain lipophilic compounds (Osorio et al. 2012). These primary metabolites are fundamental to essential physiological processes and are conserved across plant species. In contrast, LC–MS is particularly suited for detecting semi-polar metabolites associated mainly with secondary metabolism, such as alkaloids, saponins, phenolic acids, phenylpropanoids, flavonoids, glucosinolates, polyamines, glycosylated volatile organic acids, and oxylipins (de Vos et al. 2007). Secondary metabolites are often specific to species, genotypes, organs, or tissues, and may be synthesised in response to environmental conditions or developmental stages (de Vos et al. 2007; Moco et al. 2007). The application of these analytical platforms has enabled the identification of numerous metabolites in potato tubers underlying variation among varieties and transgression lines, as well as developmental stages, storage duration, cold-induced sweetening, wound healing, responses to (a)biotic stresses, and diverse growth environments (Roessner et al. 2000; Yang and Bernards 2007; Dobson et al. 2008; Shepherd et al. 2014, 2015; Uri et al. 2014; Datir et al. 2020).

To create differences in physiological ageing in tubers, in this study, we produced potato seed tubers from four contrasting cultivars on a single field in two years and stored them at different temperatures. Tuber metabolomes were assessed monthly throughout storage using GC–MS and LC–MS platforms. This paper presents the first untargeted metabolomics study, covering hundreds of metabolites, to examine multi-season datasets across the combined effects of cultivars and storage temperature in potato seed tubers. Firstly, we assessed the changes in the number of primary and secondary metabolites, detected by GC–MS and LC–MS, respectively. Next, principal component analysis (PCA) was used to assess the effects of cultivar, storage duration, and storage temperature on the metabolic profiles. Finally, factor analysis (FA) was applied to identify the major patterns of metabolic variation associated with cultivar and storage conditions. Through this approach, we aimed to provide a comprehensive view of metabolic shifts during storage, thereby advancing the understanding of physiological ageing in potato seed tubers.

## Materials and Methods

### Potato Tuber Materials and Samplings

#### Cultivars

Four cultivars were selected for their relevance to different market sectors and their contrasting physiological characteristics (Table 1). Cultivar Festien is a starch potato, while the other cultivars are grown for consumption, either as table potatoes or for processing. Cultivar Agria is one of the parental lines of cv. Lady Claire and both are considered slow-ageing cultivars based on their rate of change in physiological states. Cultivars Festien and Innovator are classified as fast-ageing cultivars (Zou et al. 2024).

**Table 1:** Cultivar information (Zou et al. 2024)

Cultivar	Year of release	Breeding company	Crossing parents	Market outlet	Maturity^1^	Dormancy^2^	Rate of ageing
Agria	1985	Agrico BV	QUARTA × SEMLO	Table/fries	5.5	7.5	Slow
Festien	2000	Averis Seeds BV	KARTEL × KA80-1920	Starch	3	7.5	Fast
Innovator	1999	HZPC Holland BV	SHEPODY × RZ84-2580	Fries	7	7	Fast
Lady Claire	1996	Meijer Potato BV	AGRIA × KW78-34–470	Crisps	7.5	7	Slow

^1^Low to high from 1 to 10 indicates late to early maturity

^2^Low to high from 1 to 10 indicates short to long dormancy

#### Tuber Materials

In 2019 and 2021, seed tubers of all four cultivars were produced in a single field at an experimental farm in Munnekezijl, the Netherlands (approx. 53˚20′ N, 6˚15′ E). In each year, the experimental production field followed a randomised block design with three blocks, resulting in a total of 12 plots (four cultivars × three blocks). Crop management adhered to standard conventional practices for certified seed potato production in the Netherlands, with strictly controlled agronomic conditions. Thermal time, expressed as the sum of daily air temperature (T-sum, ˚Cd), was calculated for the periods between planting, haulm killing, harvest, and the onset of storage treatment (see Table 2). Following harvest (dates provided in Tables 2 and 3), tubers were kept at around 15 ˚C to allow wound-healing and sorting, before certification and transport to Wageningen for storage.

**Table 2:** Dates (d/m/y) of seed tuber production from planting to storage in two seasons, and accumulated temperature-sums (T-sum, in ˚Cd) between two dates (Zou et al. 2024)

	2019/2020		2021/2022	
	Date	T-sum^1^	Date	T-sum^1^
Planting	03/05/2019	0	29/04/2021	0
Haulm killing	23/07/2019	1246	20/07/2021	1074
Harvesting	26/08/2019	663	25/08/2021	567
Start of storage treatments	15/11/2019	(1100)	05/11/2021	1042
Total		(3000)		2683

^1^Accumulated daily temperature (mean hourly air temperature above a base temperature of 0 ˚C). Estimated values in parentheses

**Table 3:** Sampling dates (d/m/y), accumulated temperature sums during storage (T-sum, in ˚Cd), and average daily temperatures (˚C) for the different storage conditions up to each sampling date. Six samplings (S0–S5) took place in 2019/2020 and eight (SH–S6) in 2021/2022. SH refers to a sampling in 2021/2022 shortly after harvest and S0 represents sampling at the onset of storage, both when no temperature treatment had been applied yet. Subsequent samplings (S1–S6) occurred after the start of storage treatment, at approximately monthly intervals. The T-sum represents the accumulated daily temperature, calculated as the average of hourly air temperature above a base temperature of 0 ˚C, from the beginning of storage to each respective sampling date. Note that in the 2019/2020 season, storage treatment began on 15/11/2019, while sampling of untreated tubers at S0 was conducted on 19/11/2019

Sampling	Date	Post-Harvest Days	Storage Days	Storage Temperatures (˚C)							
				**4**		**7**		**10**		**17**	
				T-sum	Daily	T-sum	Daily	T-sum	Daily	T-sum	Daily
**2019/2020**											
S0	19/11/2019	85	0	0	-	0	-	0	-		
S1	15/12/2019	111	30	118	3.9	223	7.4	307	10.2		
S2	21/01/2020	148	67	266	4.0	529	7.9	684	10.2		
S3	25/02/2020	183	102	400	3.9	801	7.9	1041	10.2		
S4	31/03/2020	218	137	537	3.9	1127	8.2	1398	10.2		
S5	29/04/2020	247	166	651	3.9	1343	8.1	1694	10.2		
**2021/2022**											
SH	07/09/2021	13	-	-	-			-	-	-	-
S0	05/11/2021	72	0	0	-			0	-	0	-
S1	22/11/2021	89	17	69	4.1			174	10.2	254	14.9
S2	15/12/2021	112	40	164	4.1			410	10.2	649	16.2
S3	12/01/2022	140	68	278	4.1			697	10.2	1131	16.6
S4	16/02/2022	175	103	420	4.1			1055	10.2	1732	16.8
S5	23/03/2022	210	138	556	4.0			1411	10.2	2334	16.9
S6	02/05/2022	250	178	709	4.0			1818	10.2	2963	16.6

To minimise variation in physiological age associated with tuber size (Struik and Wiersema 1999), only tubers with defined size classes were selected: 35–45 mm in the 2019/2020 season and 35–40 mm in the 2021/2022 season.

#### Storage Temperature Treatments

During each storage season, the produced seed tubers were placed in three storage rooms of approximately 10 m^2^ at the research facilities of Wageningen University & Research Centre, with temperatures and relative humidity (≈90%) under automated control. Tubers were stored in darkness, and temperatures were set at 4, 7, and 10 ˚C in 2019/2020 and, to create a wider range, at 4, 10, and 17 ˚C in 2021/2022. To account for any potential spatial variation caused by ventilation effects on temperature and relative humidity, tubers within each storage room were kept in vertically stacked open trays according to the same block design used in the production field. For detailed information during storage, we refer to Zou et al. (2024).

#### Sampling and Sample Preparation

Sampling (S) was conducted approximately monthly throughout each storage season, yielding sampling moments S0–S5 in 2019/2020 and S0–S6 in 2021/2022. In the 2021/2022 season, an additional early sampling moment (SH) was included shortly after harvest. Temperature sum and average daily storage temperatures up to each sampling moment are provided in Table 3.

At SH and S0 (prior to temperature treatment), 12 samples were collected (four cultivars × three blocks). For each subsequent sampling moment (S1–S5/S6), 36 samples were collected (four cultivars × three storage temperatures × three blocks). In total, this resulted in 192 samples in 2019/2020 and 240 samples in 2021/2022. Each sample consisted of a composite of three tubers, although in six cases in 2021/2022, only two tubers were pooled due to limited availability.

For each sample, tubers were halved lengthwise from the stolon end to the top, and one half was retained for analysis. In sprouted tubers, the apical sprout was also halved. The retained halves were then cut into small cubes, snap-frozen in liquid nitrogen, ground into a fine powder, and stored at − 80˚C until analysis. Metabolite analyses were performed after all samples from a given storage season had been collected. In the 2021/2022 season, one sample in S6 was lost during grinding.

### Metabolite Extraction and Untargeted Metabolomic Workflow

Prior to GC–MS and LC–MS analysis, 200 mg of frozen powder was weighed into Eppendorf tubes. For each analysis year, a pooled analytical quality control (QC) sample was prepared by combining powders from randomly selected samples within each storage season, followed by repeated weighing of 200 mg aliquots. Consequently, the QC sample for the 2019/2020 season differed in composition from that of the 2021/2022 season. Within each analytical series, these QC samples were used to assess analytical repeatability for each detected compound and to correct for potential variation arising from extraction batch effects, compound instability, instrumental drift, and other sources of analytical variability commonly encountered in large-scale untargeted metabolomics datasets (Wehrens et al. 2016).

#### GC–MS

For GC–MS analysis, polar (primary) metabolites were extracted from frozen, ground potato tuber powder using methanol and water, as described by Carreno-Quintero et al. (2012). Briefly, 200 mg of frozen powder was extracted with 1.4 mL of 75% (v/v) methanol in water containing 15 µg mL^−1^ ribitol (Sigma®) as an internal standard. After sonication and centrifugation (10 min each), 500 µL of the supernatant was mixed with 375 µL of chloroform (− 20 ˚C) and 750 µL of distilled H_2_O (4 ˚C). Following a second centrifugation, 50 µL aliquots of the upper (polar) phase were transferred into 180 µL glass inserts placed in a 2 mL vial. All extracts were dried overnight (16 h) by vacuum centrifugation (Savant®, SPD121P, Thermo Scientific) at room temperature. After drying, the vials were sealed under an argon atmosphere using magnetic crimp caps. Prior to analysis, dried extracts were derivatised online using a TriPlusRSH autosampler/injection robot (Thermo Scientific), as described by Garrido et al. (2021). Briefly, 17.5 µL of O-methylhydroxylamine hydrochloride (20 mg mL^−1^ in pyridine) was added to each sample and incubated for 30 min at 40 ˚C with gentle agitation. Subsequently, 17.5 µL of N-methyl-N-trimethylsilyltrifluoroacetamide (MSTFA) was added and samples were incubated for 60 min. A 10 µL alkane mixture (C10–C32) was included to determine retention indices of GC-separated metabolites. The derivatised extracts were analysed using a GC–MS system comprising a Trace 1300 gas chromatograph (Thermo Scientific) equipped with a programmable temperature vaporisation (PTV) injector and coupled to a TSQ8000 DUO-series triple quadrupole mass spectrometer (Thermo Scientific). One microlitre of each derivatised extract was injected at 70 ˚C with a split flow of 19 mL min^−1^. Chromatographic separation was performed on a VF-5 ms capillary column (Varian; 30 m × 0.25 mm × 0.25 µm), including a 10 m guard column, using helium as the carrier gas at a column flow rate of 1 mL min^−1^. The column effluent was ionised by electron impact at 70 eV, and mass spectra were acquired in full-scan mode over an m/z range of 50–600. The ion source temperature was set to 290 ˚C, and a solvent delay of 420 s was applied.

In the 2021/2022 season, nine samples intended for GC–MS analysis were lost during metabolite extraction or derivatisation.

#### LC–MS

In each Eppendorf tube, 600 μL of 99.87% methanol (MeOH) containing 0.13% formic acid was added to 200 mg frozen tuber power, resulting in an estimated final concentration of approximately 80% MeOH and 0.1% formic acid, assuming a tuber water content of ~ 80%. Metabolites were extracted and analysed using high-resolution LC–MS, as previously described (Garrido et al. 2021). Briefly, samples were sonicated for 15 min and centrifuged for 15 min, after which the protein-free supernatants were transferred to a 96-well injection plate. A 5 µL aliquot of each extract was injected, and compounds were separated by high-performance liquid chromatography (HPLC) using a Luna C18 column (2.1 × 150 mm; Phenomenex) maintained at 40 ˚C. Separation was achieved using a 45-min gradient from 5 to 35% acetonitrile in MQ water, acidified with 0.1% formic acid. The LC–MS system consisted of an Acquity HPLC (Waters) coupled to a photodiode array detector (Waters 2996) and an LTQ-Orbitrap FTMS (Thermo Scientific). Detection was performed in positive electrospray ionisation mode, scanning a mass-to-charge (m/z) range of 90–1350 at a resolution of 70,000 FWHM. QC samples were re-analysed using data-dependent MS^n^ ionisation to obtain characteristic fragment ions for the identification of compounds of interest.

##### Untargeted Pre-processing of GC–MS and LC–MS Raw Data

Raw GC–MS and LC–MS data files were independently pre-processed per storage season using Metalign software (Lommen 2009) for mass feature extraction and alignment. For the GC–MS platform, the peak detection threshold was set to 10 ion counts per scan in 2019/2020 and 100 in 2021/2022, while for the LC–MS platform, the threshold was set to 1000 for both seasons. Metalign outputs were subsequently filtered to retain features present in at least six randomly selected samples per dataset. The filtered datasets were then processed using the MSClust tool (Tikunov et al. 2012) to: (1) remove redundant mass features of compounds, and (2) generate electron impact (EI) ionisation mass spectra for GC–MS compounds and soft ionisation (full scan, ion source) mass spectra for LC–MS compounds. The minimum number of clustered mass peaks per compound (i.e., per mass spectrum) was set to five for GC–MS and two for LC–MS.

##### LC-column Leakage Issue: Alignment of Datasets from before and after Repair

In the 2021/2022 season, a slight LC solvent leak at the column connection was detected after 127 of the 273 total extracts (including QC samples) had been analysed. Following the repair, the last 10 analysed samples were re-analysed to assess whether the raw data files generated before and after the repair could still be aligned using the untargeted data processing workflow described above. A preliminary evaluation based on 20 LC–MS files suggested that re-injecting all 127 previously analysed extracts was unnecessary. However, after pre-processing all 273 LC–MS files from the 2021/2022 season, it became apparent that the mass feature alignment was suboptimal. Consequently, the raw LC–MS files acquired before and after the repair were pre-processed separately, resulting in two datasets, each containing approximately half of the samples of season 2021/2022. To merge these datasets, firstly cosine similarity scores were calculated for the mass feature clusters (ion-source mass spectra of compounds) in each data file within a three-minute retention time window using the Python package Matchms (Huber et al. 2020). Retention times of similar compounds in the two datasets were then aligned, using a moving median and Gaussian kernel approach, based on best bidirectional hits defined as cosine similarity scores above 0.6 and at least 25% higher than any other match for corresponding spectra. The aligned mass spectra from both datasets were subsequently matched using a one-minute retention time window and a cosine score threshold of 0.6, resulting in a dataset of 425 common compounds for the 2021/2022 season. Despite this alignment, principal component analysis (PCA) of the samples based on these 425 metabolites revealed a slight separation between QC samples analysed before and after the repair. This difference was attributed primarily to minor changes in chromatographic peak shapes and, consequently, peak intensities. Attempts to correct for these differences by normalisation during downstream analyses were unsuccessful and slightly increased the separation between QC samples. Therefore, unnormalised peak intensities (see supplementary data) were used in subsequent analyses. Importantly, the main biological effects on metabolome—associated with cultivar, storage temperature, and storage duration—were consistent between samples analysed before and after the leakage repair. Thus, although the leakage reduced the number of reliably aligned metabolites in the 2021/2022 dataset, it did not materially affect the overall statistical outcomes or conclusions.

Metabolite intensities were not normalised across storage seasons, as samples from each season were analysed independently and the QC samples differed between seasons, being prepared separately by pooling tuber powders within each season.

##### Metabolite Identification

For metabolites detected using GC–MS, putative annotations were based on available EI mass spectra and retention index libraries. For LC–MS data, annotation was performed manually based on the accurate mass of the putative molecular ion [M + H]^+^ within MSClust-grouped ion clusters. These molecular ions were matched, within a mass deviation of 5 ppm, to those calculated from the elemental formula of compounds previously reported in potato or other Solanaceae species (e.g., the KNApSAcK database), and where available, supported by retention time information from in-house analyses of Solanaceae samples acquired using the same LC–MS platform. However, particularly for secondary metabolites detected by LC–MS, unambiguous structural identification based solely on elemental formula is not possible in the absence of authentic standards or MS/MS fragmentation data. Therefore, metabolites were classified as unknown when multiple candidate annotations corresponded to a given elemental formula, when ion clusters were ambiguous and lacked a clearly defined molecular ion, when overlapping mass signals originating from poorly retained compounds in the injection peak, or, in the case of GC–MS, when no reliable matches were found in EI spectral libraries.

### Data Analyses

All subsequent analyses of GC–MS and LC–MS peak intensity data, including data filtering, correction, transformation, and statistical analyses, were conducted in R (R Core Team 2024).

#### Data Filtering

To enhance data reliability, metabolites that were sporadically present or exhibited frequent non-detections (i.e., values of 0) across a storage season without a clear biological basis were filtered out. Inclusion of a metabolite for further analysis was based on two criteria: (1) it was detected in all blocks at least at two consecutive sampling points within at least one cultivar × storage temperature combination, and (2) it appeared in at least seven samples across three consecutive sampling points in at least one cultivar × storage temperature combination. Additionally, metabolites absent in specific cultivars were excluded from cultivar-specific multivariate analyses, including principal component analysis (PCA) and factor analysis (FA) (supplementary data). After filtering, the number of metabolites across cultivars, storage temperatures, and sampling times was summarised using the UpSetR package to visualise intersections.

#### Data Treatment for Non-Detects

Following the filtering of unreliable metabolites, the remaining datasets still contained non-detects (zeros), which pose challenges for multivariate analyses. These non-detects might have arisen from three potential sources: (1) true absence of the metabolite (i.e., the metabolite was not detectable with current analytical instruments; e.g., cultivar-specific metabolites), (2) low abundance below the limit of peak detection (LOD) for the respective platform (as described earlier), or (3) analytical or preprocessing issues resulting in the exclusion of detectable metabolites. In Case 1, no correction was needed, as the zeros reflected a genuine absence. In Case 2, zeros could be replaced with values below the LOD. In Case 3, zeros might be treated as missing values and imputed using appropriate statistical methods (Wei et al. 2018). However, distinguishing among these cases was practically infeasible. Therefore, all zeros were replaced with a randomised value between 40 and 60% of the minimum detected relative abundance for each metabolite. This approach introduces limited variability and improves upon the conventional fixed half-minimum (50%) imputation.

#### Data Transformation

After filtering and imputing non-detects, metabolite intensity values were log10-transformed to reduce heteroscedasticity and stabilise variance (van den Berg et al. 2006).

#### Multivariate Analyses

Two unsupervised multivariate analysis methods were applied: principal component analysis (PCA) and factor analysis (FA).

PCA was performed separately on the GC–MS and LC–MS datasets for each storage season, both across all cultivars and for each cultivar individually. Analyses were conducted using the ggfortify package, with centring and auto-scaling applied for data standardisation.

Factor analysis was performed on the combined GC–MS and LC–MS datasets for each storage season, as well as for individual cultivars within each season. The analysis was conducted using an automated implementation (Navarro 2023) of the regularised maximum likelihood approach in the R package FMradio (Peeters et al. 2019). To reduce collinearity, redundancy filtering was applied, retaining one metabolite from each pair of equivalent (variables identified metabolite pairs are listed in the supplementary data). The optimal number of latent variables (i.e., factors) was determined using the Guttman bound, ensuring that only factors explaining variance beyond that attributed to unique variance were retained. Factor loadings were computed to quantify the contribution of each metabolite to the extracted factors. These loadings were subsequently used to calculate both factor-explained variances and factor scores. Factor-explained variance was calculated as the sum of squared loadings across all metabolites for each factor, representing the proportion of total variance explained. Factor scores, representing the position of each sample along each factor, were calculated as weighted linear combinations of metabolite intensities, with factor loadings used as weights (Peeters et al. 2019). Metabolites with absolute factor loadings greater than 0.3 were considered significant contributors to their respective factors (Navarro 2023).

## Results

### Number of Metabolites

The number of analysed samples is provided in Table S1. Untargeted data pre-processing (see Materials and Methods) yielded a total of 1218 metabolites in the 2019/2020 storage season, comprising 113 detected by GC–MS and 1105 by LC–MS. In comparison, 564 metabolites were detected in 2021/2022, including 139 GC–MS and 425 LC–MS metabolites (Table S2). The relatively low number of LC–MS metabolites in the second season was mainly attributable to the analytical issue discussed above. After data filtering to retain only reliably detected compounds (see Materials and Methods), 933 metabolites remained for the 2019/2020 season (104 GC–MS and 829 LC–MS), while 492 metabolites were retained for 2021/2022 (117 GC–MS and 375 LC–MS) (Table S2). Subsequent analyses were based on these filtered datasets.

In both seasons, all GC–MS metabolites were detected at least once in each cultivar, whereas several LC–MS metabolites were absent in specific cultivars, with some appearing to be cultivar-specific (Figs. S1–S2). A similar pattern was observed across storage temperatures, where all GC–MS metabolites were detected at least once at each temperature, whereas a small number of LC–MS metabolites were absent at 4 ˚C (Figs. S3–S4). Over the storage period, GC–MS metabolites were consistently detected at all sampling points, whereas several LC–MS metabolites were absent during early storage stages and others appeared only at later stages (Figs. S5–S6).

**Fig. 1:**
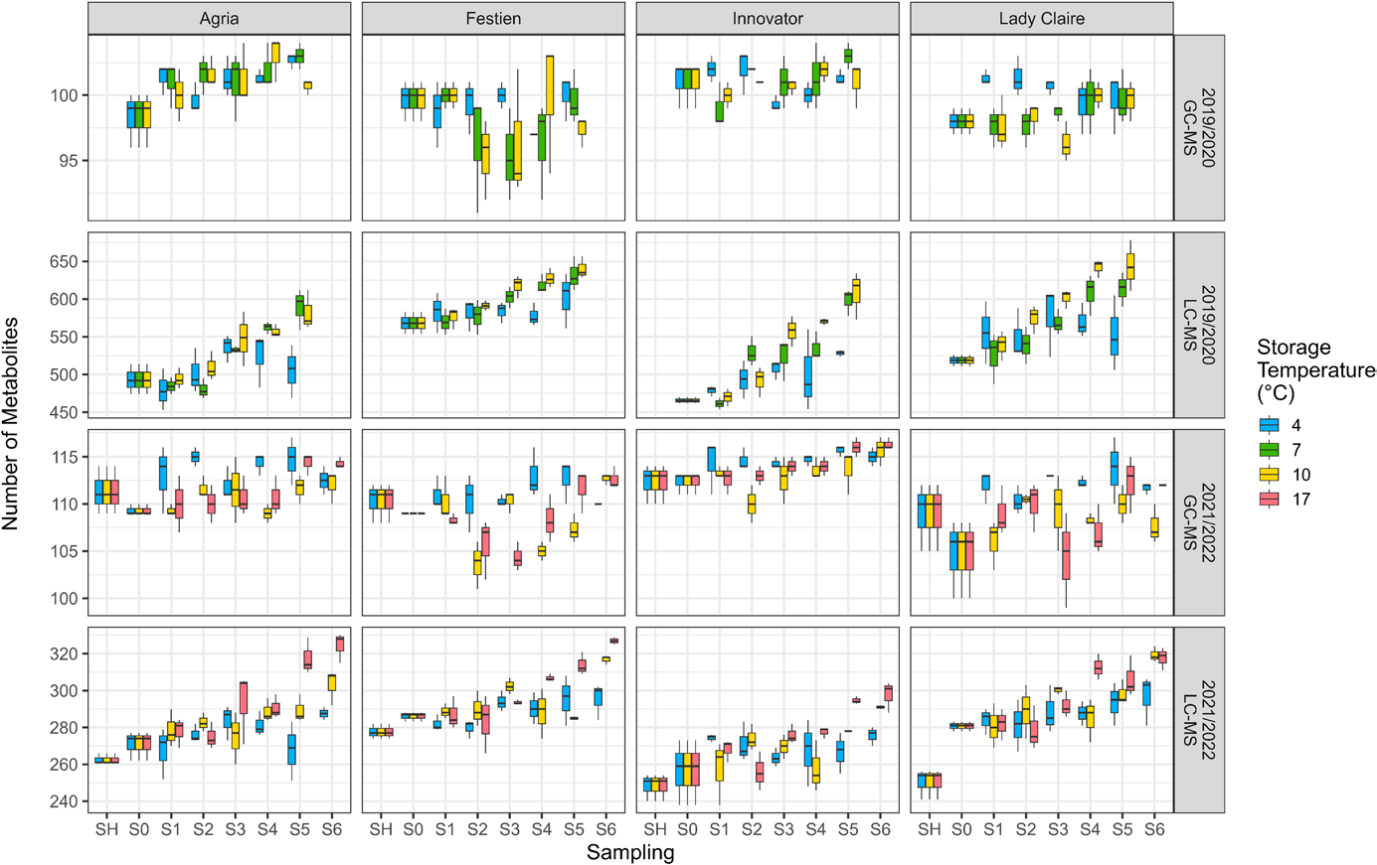
Number of GC–MS and LC–MS metabolites (after data filtering) in four cultivars, measured before (SH and S0) and during (S1—S6) storage at different temperatures (indicated by colours) across two storage seasons (2019/2020: S0—S5, first and second rows; 2021/2022: SH—S6, third and fourth rows). Each box plot represents values from up to three blocks. Boxes indicate the interquartile range (IQR), with the central line representing the median, and whiskers extending to the most extreme values within 1.5 × IQR from the lower and the upper quartiles. Boxes for SH and S0 are duplicated across temperatures

Significant differences among cultivars were observed in the number of GC–MS metabolites (Fig. 1, Table S3). Within cultivars, in 2019/2020, no significant interaction between storage temperature and duration was detected; however, significant main effects were observed for storage duration in cv. Agria, and for storage temperature in cv. Lady Claire. In 2021/2022, both storage duration and temperature had significant effects across all cultivars, with a significant interaction observed in cv. Festien (Table S4). These differences were generally associated with higher numbers of metabolites detected at 4 ˚C compared with higher temperatures, and at later stages compared with early stages of storage.

The number of LC–MS metabolites also differed significantly among cultivars and increased markedly during storage and at higher temperatures (Fig. 1, Table S3). Prior to storage treatment (SH and/or S0), cv. Festien exhibited the highest number of LC–MS metabolites among cultivars, whereas tubers from the other cultivars stored at higher temperatures gradually reached comparable levels. Within cultivars, in addition to significant main effects of storage temperature and duration, a significant interaction between these factors was observed in all cultivar-season combinations except cv. Festien in 2019/2020 (Table S5). Notably, the number of LC–MS metabolites increased between SH (shortly after harvest) and S0 (prior to storage treatment) in 2021/2022, particularly in cv. Lady Claire (Fig. 1).

### Multivariate Analyses

#### PCA Indicates Key Roles of Cultivar, Storage Temperature, and Storage Duration in the Tuber Metabolome

**Fig. 2:**
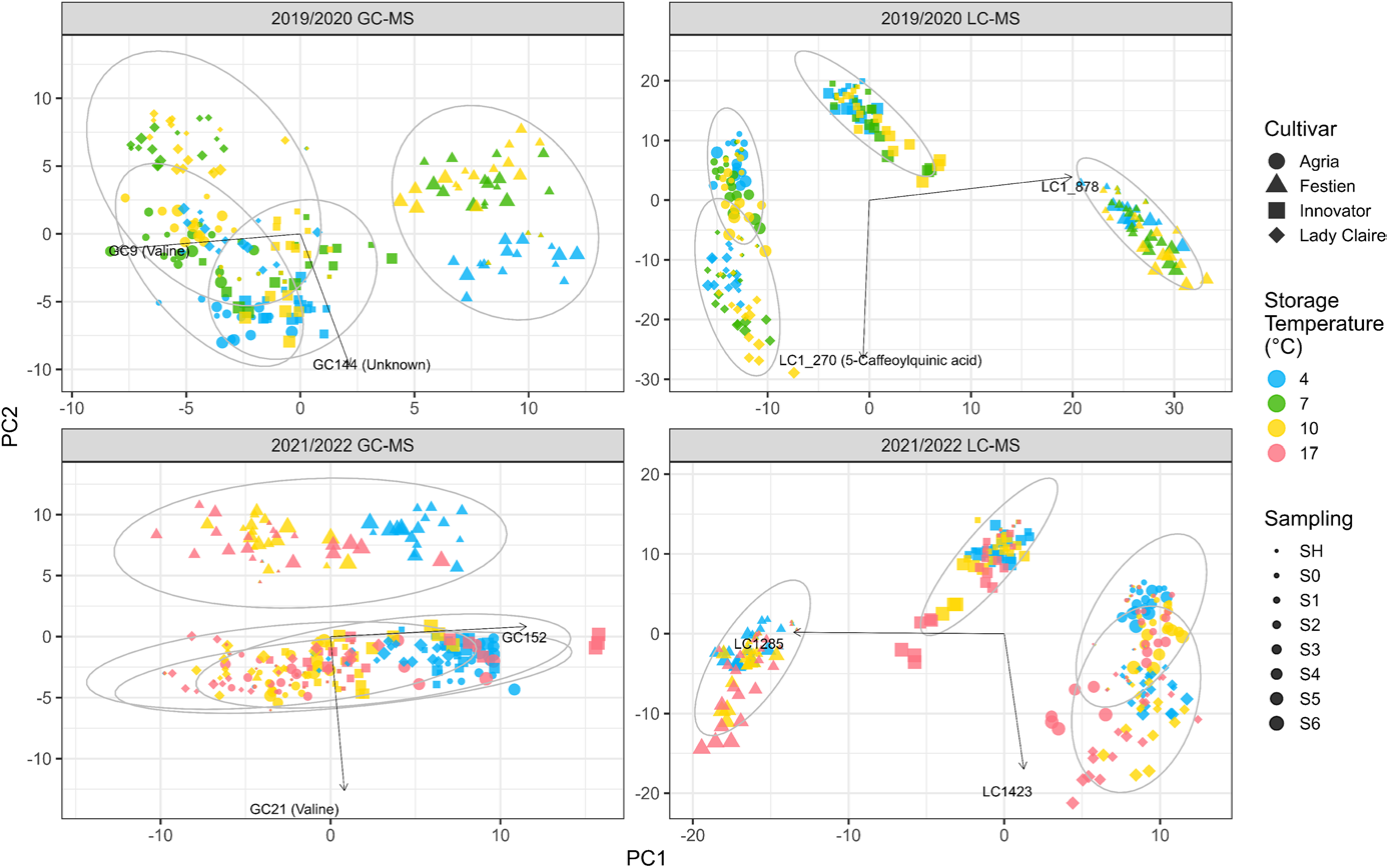
PCA score plots based on GC–MS (left) and LC–MS (right) metabolites for the 2019/2020 (top) and 2021/2022 (bottom) storage seasons. Each point represents a sample, with cultivars indicated by shape (with 95% confidence ellipses), storage temperatures by colour, and sampling by increasing size (from early to late). Arrows point to the metabolites with the highest absolute loadings on PC1 and PC2 and their putative annotations are listed when available. The variances explained by PC1 and PC2 are: 2019/2020 GC–MS (28.7% and 17.6%), 2019/2020 LC–MS (32.4% and 16.5%), 2021/2022 GC–MS (23.5% and 18.4%), 2021/2022 LC–MS (28.3% and 16.3%)

**Fig. 3:**
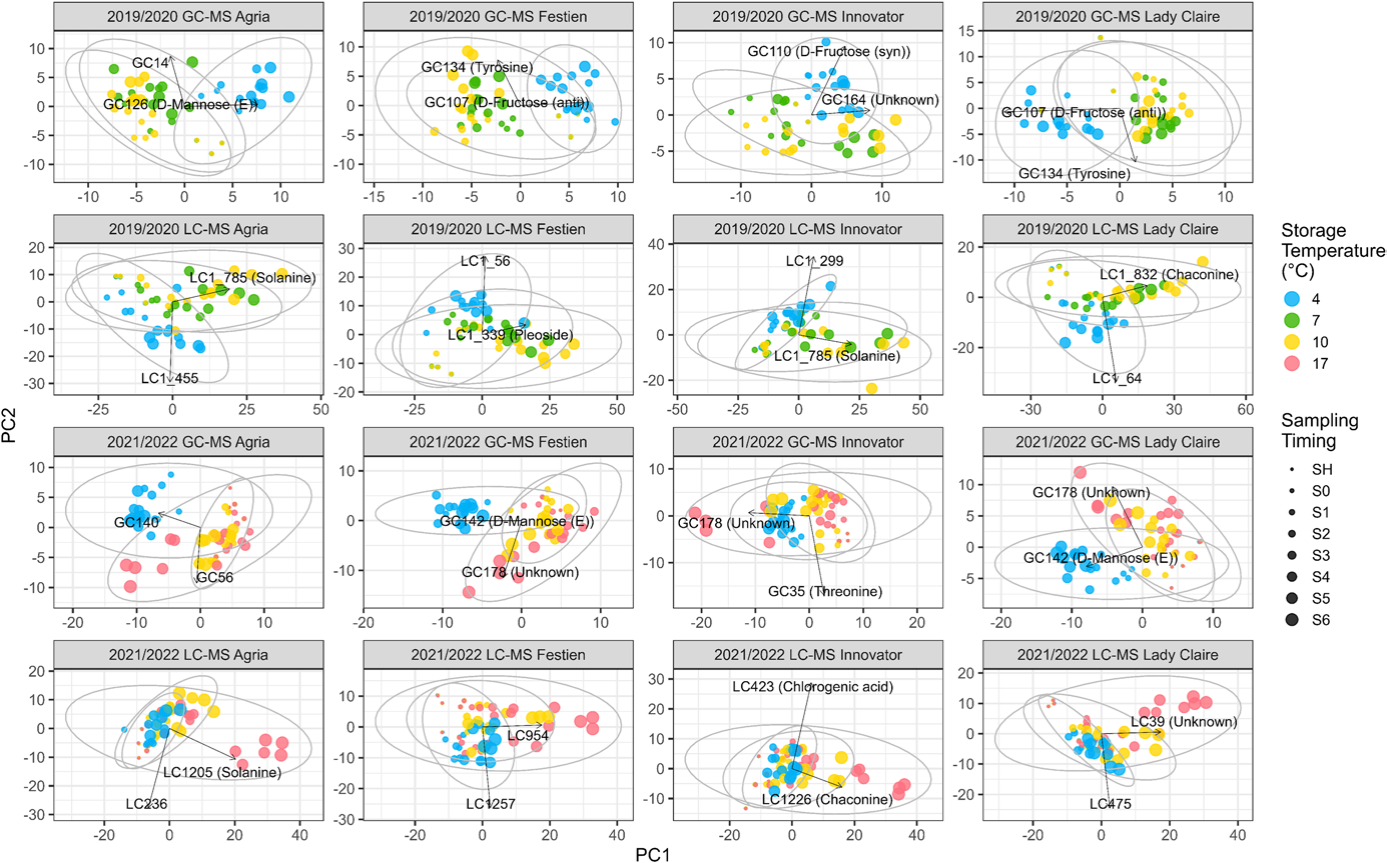
PCA score plots for each cultivar based on GC–MS (first and third row) and LC–MS (second and fourth row) metabolites for the 2019/2020 (first two rows) and 2021/2022 (last two rows) storage seasons. Each point represents a sample, with storage temperatures indicated by colour (with 95% confidence ellipses) and sampling by increasing size (increasing from early to late). Arrows point to the metabolites with the highest absolute loadings on PC1 and PC2 and their putative annotations are listed when available. The variance explained by PC1 and PC2 is: 2019/2020 GC–MS—cv. Agria (26.9%, 16.6%), cv. Festien (25.9%, 13.3%), cv. Innovator (27.6%, 11.9%), cv. Lady Claire (20.7%, 20.4%); 2019/2020 LC–MS—cv. Agria (26.7%, 8.7%), cv. Festien (26.5%, 8.2%), cv. Innovator (33.5%, 8.7%), cv. Lady Claire (33.4%, 8.0%); 2021/2022 GC–MS—cv. Agria (32.8%, 13.9%), cv. Festien (25.4%, 14.6%), cv. Innovator (34.3%, 11.1%), cv. Lady Claire (27.7%, 13.2%); 2021/2022 LC–MS—cv. Agria (29.5%, 10.1%), cv. Festien (30.0%, 10.6%), cv. Innovator (33.9%, 8.1%), cv. Lady Claire (30.8%, 10.7%)

PCA was performed separately on GC–MS and LC–MS datasets for the 2019/2020 and 2021/2022 storage seasons. PCA score plots were generated using the first two principal components (PC1 and PC2) for all four datasets (Fig. 2), as well as for cultivar-specific analyses (Fig. 3).

For GC–MS metabolites (Fig. 2, left), in 2019/2020, PC1 separated cv. Festien from the other three cultivars, while PC2 distinguished tubers stored at 4 ˚C from those stored at higher temperatures. In 2021/2022, these patterns were largely reversed, likely reflecting a stronger temperature effect due to the wider temperature range (4, 10, 17 ˚C) compared with 2019/2020 (4, 7, 10 ˚C). In both seasons, samples stored at higher temperatures gradually shifted towards those stored at 4 ˚C, particularly in cv. Innovator. Valine showed the highest absolute loading on PC1 in 2019/2020 and on PC2 in 2021/2022.

In the cultivar-specific GC–MS PCA (Fig. 3, first and third rows), PC1 generally captured the contrast between storage at 4 ˚C and higher temperatures, whereas PC2 reflected temporal progression. However, in cv. Innovator (both seasons) and cv. Lady Claire (in 2021/2022), these roles were reversed. Where annotated, the highest positive loadings on PC1 were typically associated with sugars, such as mannose and fructose, while PC2 was mainly driven by amino acids; for example, tyrosine showed the highest absolute loadings in cvs. Festien and Lady Claire in 2019/2020, and threonine in cv. Innovator in 2021/2022.

For LC–MS metabolites (Fig. 2, right), PC1 primarily captured the cultivar differences in both seasons, with cvs. Festien and Innovator clearly separated from the other cultivars. The partial overlap between cvs. Agria and Lady Claire may reflect their genetic relationship, as cv. Agria is a crossing parent of cv. Lady Claire (Table 1). PC2 showed a consistent trajectory associated with storage duration (early to late) and temperature (cold to warm). In 2021/2022, 5-caffeoylquinic acid had the highest loading on PC2.

In the cultivar-specific LC–MS PCA (Fig. 3, second and fourth rows), the first two PCs suggested an interaction between storage duration and temperature, particularly in 2021/2022. Across cultivars in both seasons, the highest loadings on PC1 were most often attributed to glycoalkaloids (where annotated), including solanine and chaconine. An exception was cv. Festien in 2019/2020, where pleoside showed the highest loading on PC1.

#### FA: Top Factors Indicate Dominant Patterns of Metabolic Shifts

To uncover latent patterns of variation across a broad range of metabolites, FA was conducted separately for each storage season using the combined GC–MS and LC–MS datasets. In both seasons, 12 factors were computed (Table 4), summarising the principal patterns of metabolic variation. In each season, Factor 1 (F1) explained the largest proportion of total variance and was associated with more than half of all metabolites (Table 4). Across seasons, the first four to six factors captured the majority of metabolites, whereas the remaining factors each explained less than 1% of the total variance and were associated with relatively few metabolites (Table 4).

**Table 4:** Factor-explained variance (% of total) and the corresponding number of metabolites associated with absolute loadings > 0.3

Season	Factor	F1	F2	F3	F4	F5	F6	F7	F8	F9	F10	F11	F12
**2019/2020**	Variance	28.6%	16.2%	7.8%	4.4%	1.0%	0.7%	0.2%	0.2%	0.1%	0.1%	0.1%	0.1%
	Number	633	446	222	138	22	15	0	4	2	1	0	0
**2021/2022**	Variance	33.6%	18.1%	10.0%	6.1%	5.6%	4.6%	0.9%	0.7%	0.4%	0.3%	0.3%	0.2%
	Number	340	248	131	98	67	64	3	8	0	1	1	2

**Fig. 4:**
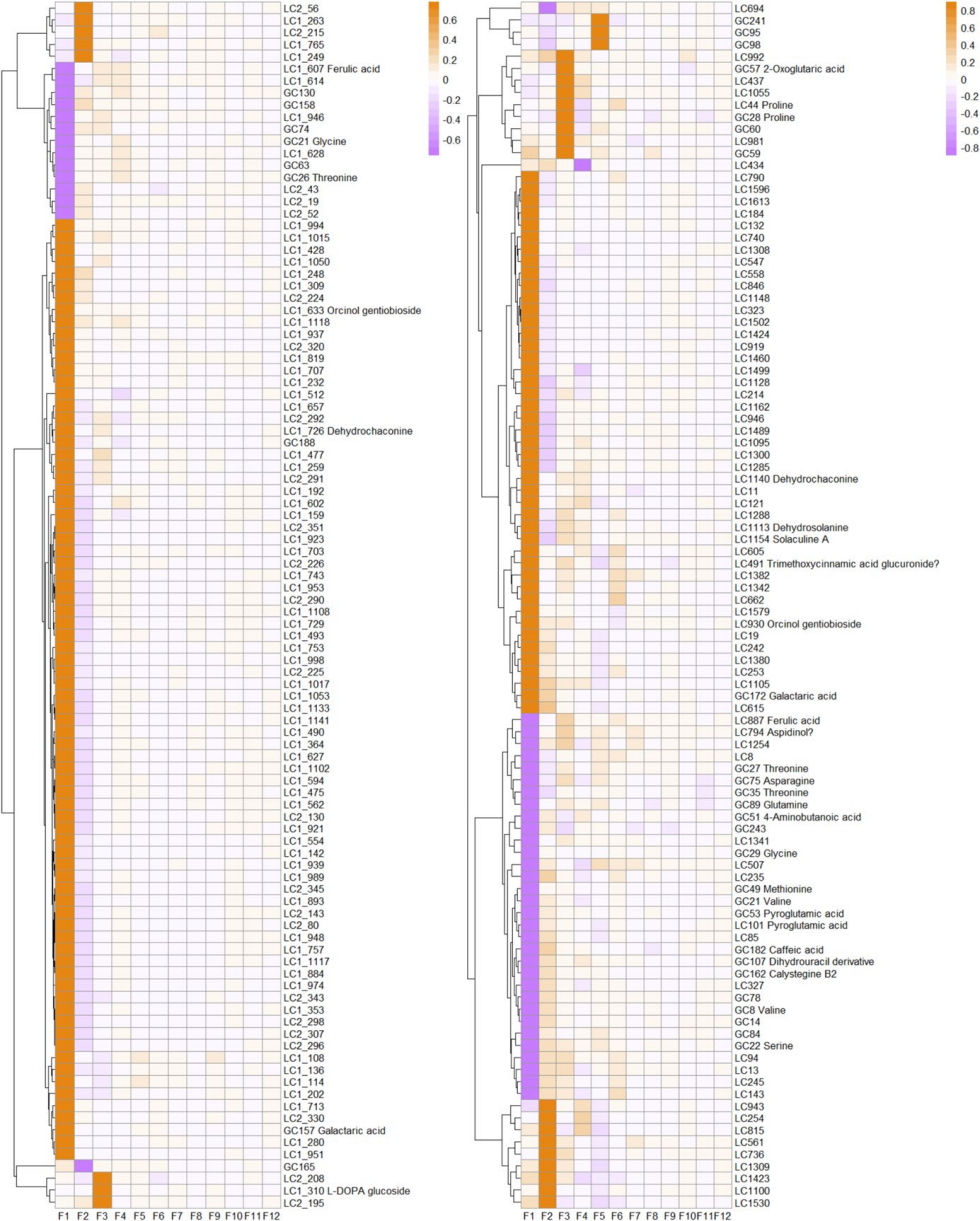
Heatmap of factor loadings for the top 100 metabolites with the highest absolute loadings across all factors in the 2019/2020 (left) and 2021/2022 (right) storage seasons. Columns represent factors, and rows correspond to metabolites, which are organised by hierarchical clustering. The colour gradient—from dark purple (high negative loadings), through white (zero loading), to dark orange (high positive loadings)—represents loading values ranging from − 1 to 1. Putative metabolite annotations, where available and considered reliable, are shown alongside metabolite cluster IDs; low-confidence annotations are indicated by “?”. In the 2021/2022 dataset (right), GC27 and GC35 correspond to threonine (detected as threonine-2TMS and threonine-3TMS, respectively), while GC8 and GC21 correspond to valine (detected as valine-TMS and valine-2TMS, respectively), reflecting different GC–MS derivatisation forms of the same compound. In addition, some metabolites were detected by both GC–MS and LC–MS platforms; for example, GC28 and LC44 correspond to proline, and GC53 and LC101 to pyroglutamic acid

In each storage season, the top 100 metabolites ranked by absolute loadings were visualised in a heatmap, where metabolites were grouped by hierarchical clustering and factor loadings were presented by colour gradients (Fig. 4). Putative annotations, when available, were displayed alongside cluster IDs. Most GC–MS metabolites could be annotated, whereas LC–MS metabolites were largely unannotated (see Materials and Methods). In 2019/2020, most metabolites showed strong positive loadings on F1, with smaller groups exhibiting negative loadings on F1 or positive loadings on F2 and F3. Loadings on the remaining factors were generally low, mostly below 0.3 in absolute values. A similar pattern was observed in 2021/2022: the majority of metabolites were positively associated with F1, accompanied by subsets negatively associated with F1 or positively associated with F2 and F3, and a small number of GC–MS metabolites were positively associated with F5. Across both seasons, metabolites strongly and positively associated with F1 were predominantly detected by LC–MS, whereas most GC–MS metabolites showed negative associations with F1. Among the annotated metabolites, ferulic acid was negatively associated with F1 and, to a lesser extent, positively associated with F3 in both seasons. Glycine and threonine were consistently negatively associated with F1, whereas orcinol gentiobioside, dehydrochaconine, and galactaric acid showed positive associations with F1 across seasons (Fig. 4). Season-specific patterns were also evident. In 2019/2020, L-DOPA glucoside showed a positive loading on F1. In 2021/2022, dehydrosolanine and solaculine A were positively associated with F1, while asparagine, glutamine, 4-aminobutanoic acid, methionine, valine, caffeic acid, a dihydrouracil derivative, calystegine B2, and serine exhibited negative loadings on F1. Additionally, 2-oxoglutaric acid and proline showed positive loadings on F3.

**Fig. 5:**
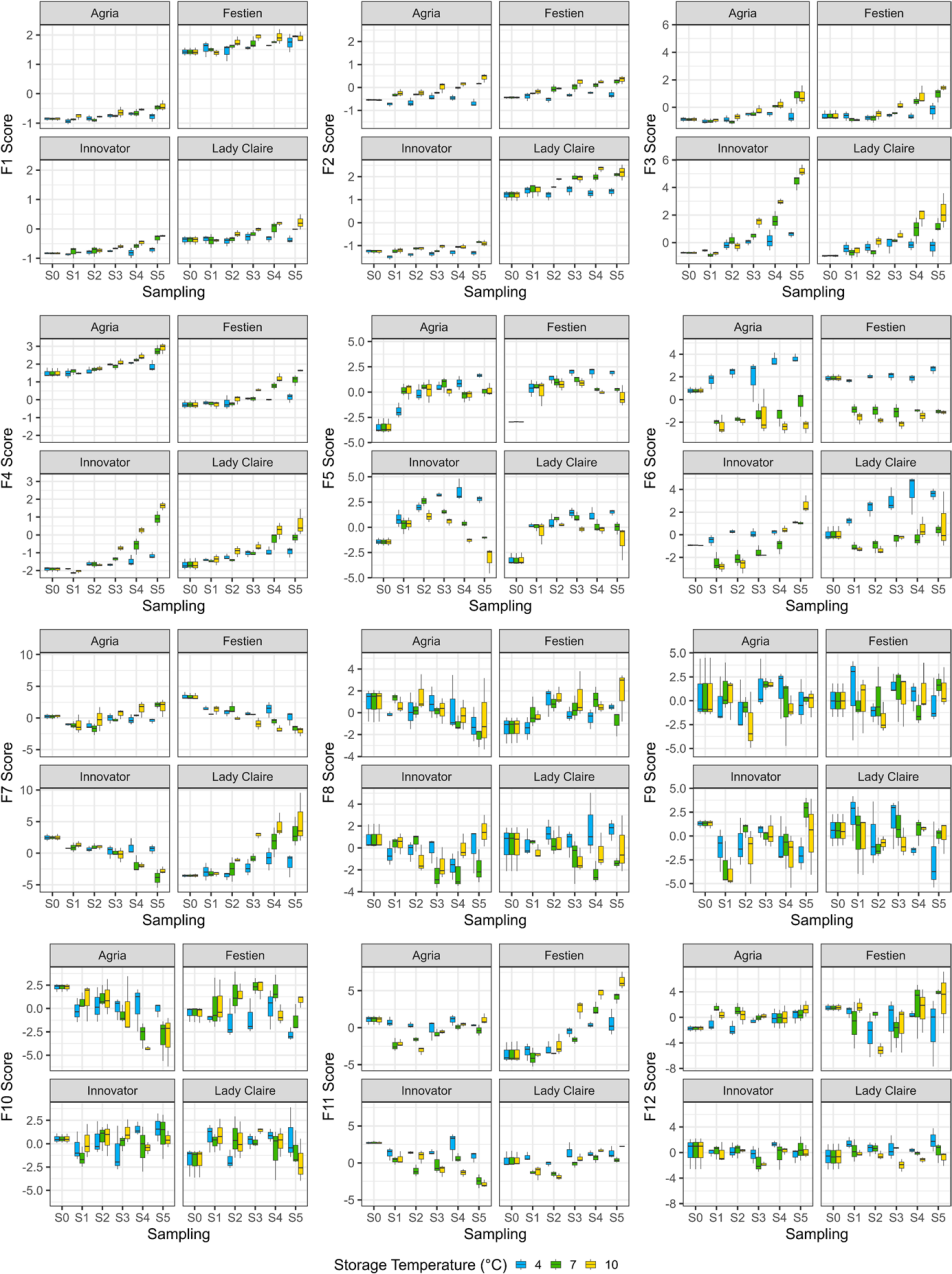
Factor scores of four cultivars stored at three temperatures (in colours) and sampled across six sampling time points in the 2019/2020 storage season. Each box plot represents values from up to three blocks, where boxes indicate the interquartile range (IQR), with the central line representing the median, and whiskers extending to the most extreme values within 1.5 × IQR from the lower and the upper quartiles

**Fig. 6:**
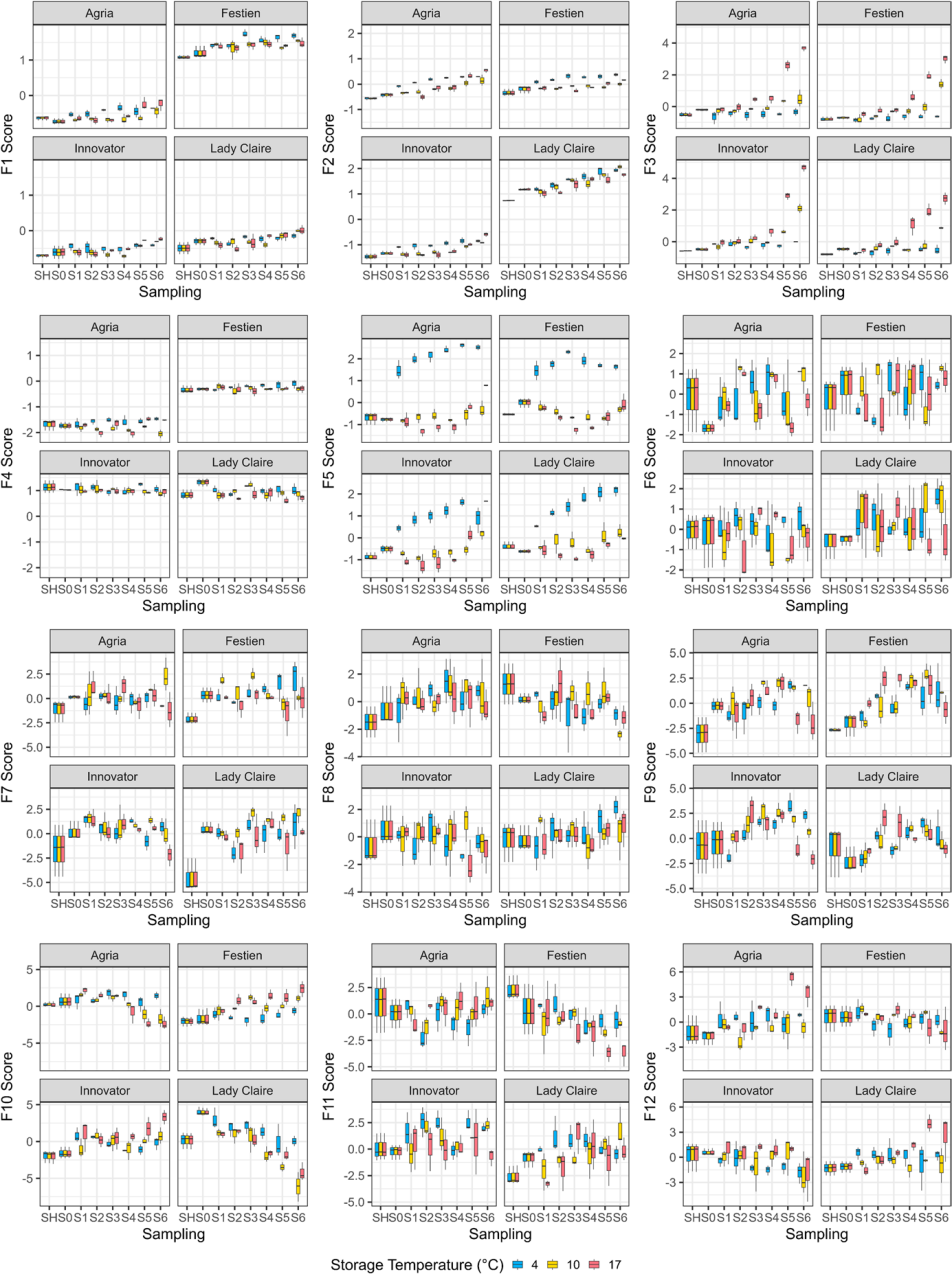
Factor scores of four cultivars stored at three temperatures (in colours) and sampled across eight sampling time points in the 2021/2022 storage season. Each box plot represents values from up to three blocks, where boxes indicate the interquartile range (IQR), with the central line representing the median, and whiskers extending to the most extreme values within 1.5 × IQR from the lower and the upper quartiles

To visualise the latent variables, factor scores—representing sample positions on each factor as weighted linear combinations of metabolite peak intensities (using the factor loadings as weights)—were plotted against sampling time, with panels arranged by cultivar and storage temperatures indicated by colour (Figs. 5, 6). In the 2019/2020 season, the top four factor scores generally increased over time and at higher storage temperatures, although patterns varied across cultivars. For F1, cv. Festien consistently exhibited higher scores than the other cultivars. In F2, cvs. Innovator and Lady Claire displayed contrasting profiles. F3 showed clear increases over time at higher temperatures across all cultivars, particularly in cv. Innovator, while F4 followed a similar trend, with cv. Agria contrasting against cvs. Innovator and Lady Claire. Among subsequent factors, F5 showed an initial increase followed by a plateau at lower temperatures or a decline at higher temperatures. F6 captured strong effects of cold storage (4 ˚C), although this effect diminished towards the end of storage in cv. Innovator. F7 decreased over time in cvs. Festien and Innovator but showed increasing trends in cvs. Agria and Lady Claire. Lower-ranked factors exhibited greater variability and no clear patterns with respect to cultivar, time, or temperature (Fig. 5). In 2021/2022, several factors showed correspondence with those from the previous season: F1–F3 aligned closely with their counterparts, while F4 showed partial similarity despite weaker time and temperature trends. In addition, F5 resembled F6, and F10 resembled F7 (with reversed trends). As before, lower-ranked factors were more variable and less interpretable; however, F7 and F9 displayed patterns similar to F5 from 2019/2020, characterised by an initial increase followed by a plateau or decline (Fig. 6).

The top factors from both seasons revealed strong cultivar effects. To disentangle these from storage treatment effects, FA was also performed separately for each cultivar. In these cultivar-specific datasets, more than half of the metabolites loaded onto F1, and most were associated with the top five to six factors (Table S6). Factor score plots showed that F1 consistently increased with higher storage temperatures across all cultivars (Figs. S7–S14). The effects of cold storage (4 ˚C) were particularly pronounced in specific factors depending on cultivar: in 2019/2020, these were in F3 in cv. Agria (Fig. S7), F2 in cv. Festien (Fig. S8), F5 in cv. Innovator (Fig. S9), and F3 in cv. Lady Claire (Fig. S10), and in 2021/2022, F3 in cv. Agria (Fig. S11), F4 and F5 in cv. Festien (Fig. S12), F3 in cv. Innovator (Fig. S13), and F5 in cv. Lady Claire (Fig. S14). Several factors displayed an initial increase followed by a plateau at lower temperatures or a decline at higher temperatures. This pattern was observed in 2019/2020 for F2 in cv. Agria (Fig. S7), F3 in cv. Festien (Fig. S8), F3 in cv. Innovator (Fig. S9), and F2 in cv. Lady Claire (Fig. S10), and in 2021/2022 for F4 in cv. Agria (Fig. S11), F5 in cv. Festien (in reverse) (Fig. S12), and F4 in cv. Innovator (Fig. S13) in 2021/2022. The remaining top-ranked factors did not show clear responses to storage treatments.

## Discussion

Metabolic activity in potato tubers during storage, largely driven by physiological ageing, is highly relevant to both the food industry, where it influences product quality, and the seed potato sector, where it affects seed vigour and subsequent crop performance (van der Zaag and van Loon 1987; Pinhero et al. 2009; Zou et al. 2025). Previous studies on metabolites involved in storage and physiological ageing have mainly relied on targeted analysis of well-established compounds. Metabolomics has emerged as a promising approach for detecting subtle, continuous changes in tubers and for linking phenotypic variation to underlying genotypic differences (Fernie and Schauer 2009; Saito and Matsuda 2010). In this context, GC–MS platforms have enabled a growing number of untargeted studies (e.g., Roessner et al. 2001; Beckmann et al. 2007; Shepherd et al. 2007; Carreno-Quintero et al. 2012; Uri et al. 2014; Fukuda et al. 2019; Villányi et al. 2022), supported by extensive universal mass spectral libraries that incorporate GC retention indices (Hall 2006; Wehrens and Salek 2019). More recently, LC–MS has been increasingly used to explore secondary metabolites that were previously largely inaccessible (e.g., Shepherd et al. 2010, 2014, 2015; Chong et al. 2013; Claassen et al. 2019), although compound annotation remains limited due to the relatively underdeveloped nature of LC–MS spectral libraries and the variability of retention times across laboratories. Because GC–MS and LC–MS are largely complementary, their combined application enables more comprehensive coverage of the plant metabolome. In this study, the combined use of GC–MS and LC–MS platforms enabled the detection, after data filtering, of 933 and 492 metabolites in the 2019/2020 and 2021/2022 storage seasons, respectively. This extensive dataset, together with an experimental design incorporating multiple cultivars, storage temperatures, and sampling time points, provided a robust basis for identifying major metabolic changes associated with physiological ageing during storage. Although the second season included a higher number of sampling points (eight versus six) and a broader range of storage temperatures (4, 10, 17 ˚C versus 4, 7, and 10 ˚C), only approximately half the number of metabolites was detected (Fig. 1). This reduction was primarily due to analytical challenges with the LC–MS platform: an initially undetected solvent leak caused chromatographic shifts and reduced signal intensities, leading to fewer reliably aligned features after data processing. Despite these limitations, the robustness and reproducibility of the overall approach were supported by consistent patterns of cultivar and storage treatment effects across seasons (Figs. 2, 3, 5, 6). Further improvements in analytical performance would benefit from analysing all samples from both storage seasons within a single batch. In addition, metabolite identification would be enhanced by the use of high-resolution MS/MS instrumentation and advanced tools for automated spectral matching to facilitate compound annotation and biochemical pathway classification (e.g., van der Hooft et al. 2023), as well as by the continued development of comprehensive MS/MS spectral libraries.

The number of both GC–MS and LC–MS metabolites differed significantly among cultivars, highlighting a strong genotypic influence on tuber metabolome and the importance of cultivar selection in shaping outcomes (Fig. 1, Table S3). While some LC–MS metabolites were absent under specific cultivar, temperature, or storage-duration conditions, all GC–MS metabolites were detected at least once across these groups (Figs. S1–S6). This likely reflects the relatively stable presence of primary metabolites, which were predominantly detected by GC–MS, in contrast to secondary metabolites, mainly detected by LC–MS, whose synthesis is often cultivar-specific and restricted to developmental stages or environmental conditions (de Vos et al. 2007). Within cultivars, both storage temperature and duration exerted significant main effects on the number of GC–MS metabolites, indicating relatively independent influences on primary metabolism. In contrast, significant interactions between storage temperature and duration were observed for the number of LC–MS metabolites across all cultivars (Tables S4–S5), suggesting a stronger involvement of secondary metabolites in the progression of physiological ageing. Notably, variation in the number of LC–MS metabolites was already evident between SH and S0 in 2021/2022, prior to the application of storage treatments, indicating ongoing biochemical activity during early physiological stages. Such post-harvest, pre-storage metabolic dynamics in tuber remain poorly characterised, yet they may play a critical role in shaping subsequent storage-related ageing processes and overall seed tuber quality.

Multivariate analyses provided valuable insights into major metabolic shifts in potato seed tubers during storage. While both PCA and FA are widely used for dimensionality reduction in large datasets, PCA identifies linear combinations of variables that capture the maximum variation in the data, whereas FA explains covariation among variables through a small number of latent factors (Finch 2020; Peeters et al. 2019). In this study, PCA was applied to assess the effects of cultivar and storage on tuber metabolic profiles (Figs. 2, 3), and FA was used to identify latent variables representing common patterns of metabolic changes across a wide range of metabolites during storage (Figs. 5, 6). Notably, the leading principal components revealed clear cultivar differences in both GC–MS and LC–MS datasets, particularly distinguishing cv. Festien—the only starch potato cultivar—from the other cultivars (Fig. 2). Consistently, Factor 1 (F1) in the FA indicated a higher relative metabolite abundance in cv. Festien compared with the other cultivars (Figs. 5, 6), with most metabolites in both seasons strongly associated with this factor (Table 4). This could suggest a fundamentally distinct metabolic profile in the starch potato cultivar compared with consumption cultivars. Although detailed characterisation of this difference remains limited, previous work has reported a stronger influence of genetic factors than environmental conditions on the potato tuber metabolome Fukuda et al. (2019).

Among the GC–MS metabolites, PC1 in 2019/2020 and PC2 in 2021/2022 separated cv. Festien from the other cultivars, with valine showing the highest loading on these components, indicating comparatively lower valine levels in cv. Festien (Fig. 2). This pattern was further supported by the heatmap (Fig. 4), in which valine was negatively associated with F1 in 2021/2022, a factor corresponding to high metabolite abundance in cv. Festien (Fig. 6). In the cultivar-specific PCAs, tyrosine exhibited the highest loading on PC2 in both cvs. Festien and Lady Claire in 2019/2020, suggesting an increase in tyrosine levels with storage duration (Fig. 3). In contrast, in 2021/2022, threonine showed the highest loading on PC2 in cv. Innovator, indicating a decline in threonine over time in this cultivar (Fig. 3). Consistently, FA revealed negative associations between threonine and F1 across both seasons (Fig. 4), suggesting lower threonine abundance in cv. Festien and overall decline during storage. Similarly, ferulic acid and glycine were negatively associated with F1 across seasons (Fig. 4). Collectively, these patterns point to distinct amino acid profiles among cultivars and dynamic changes over the storage period.

Storage temperatures also contributed substantially to variation among GC–MS metabolites, particularly by separating tubers stored at 4 ˚C from those stored at higher temperatures. This separation was evident along PC2 in 2019/2020 and PC1 in 2021/2022 (Fig. 2), as well as along PC1 in most cultivar-specific PCA plots, and was largely driven by (stereoisomers of) mannose and fructose (Fig. 3). These patterns reflect cold-induced sweetening, a process in which starch is converted into reducing sugars during low-temperature storage. Notably, in 2021/2022, tuber samples of cv. Innovator stored at higher temperatures, particularly at 17 ˚C, displayed PCA patterns that gradually approached and eventually exceeded those of tubers stored at 4 ˚C along the corresponding PC1 axis by the end of storage (Figs. 2, 3). Consistent with this observation, FA identified latent variables (F6 in 2019/2020 and F5 in 2021/2022) associated with high metabolite abundance predominantly under cold-storage conditions; however, in cv. Innovator, tubers stored at higher temperatures exceeded these levels toward the end of storage (Figs. 5, 6). These patterns likely reflect sugar accumulation associated with tuber ageing and sprouting (Shepherd et al. 2010; Datir et al. 2020), consistent with the pronounced sprouting observed in cv. Innovator during storage and subsequent testing conditions (Zou et al. 2024).

In the full PCA, LC–MS metabolites exhibited clear cultivar-dependent variation, contributing primarily to PC1 in both seasons, while PC2 reflected progression with increasing storage duration and temperature (Fig. 2). In 2019/2020, 5-caffeoylquinic acid, a chlorogenic acid, showed the highest loading on PC2. In the cultivar-specific PCAs, PC1 was predominantly driven by solanine and chaconine, whereas in cv. Festien (2019/2020), pleoside (also known as domesticoside) contributed most strongly (Fig. 3). These patterns suggest that glycoalkaloids and glycosides are strongly associated with increasing storage temperature and duration, and may therefore be linked to physiological ageing. FA further supported these observations, with the dominant factor (F1) being largely associated with LC–MS metabolites (Fig. 4). Among these, dehydrochaconine, a minor glycoalkaloid derived from chaconine, consistently showed strong positive loadings on F1 across both storage seasons. Glycoalkaloids are known to accumulate in potato sprouts (Shepherd et al. 2010; Deng et al. 2021), and their strong association with F1 likely reflects increased sprouting during storage. Consistent with this interpretation, F1 indicated higher relative abundance in cv. Festien, a starch cultivar reported to contain high levels of total steroidal glycoalkaloid (766.20 mg/kg fresh weight) (Li et al. 2024). In addition, orcinol gentiobioside was positively associated with F1 in both seasons (Fig. 4). As with other glycosides, its functional role in tuber physiological ageing remains unclear and warrants further investigation.

Other factors were associated with fewer metabolites and explained a smaller proportion of the total variance. Among the top 100 metabolites, only a limited number of LC–MS metabolites were linked to F2, whereas both LC–MS and GC–MS metabolites contributed to F3 in 2021/2022 (Fig. 4). In addition, several factors captured notable dynamic patterns. For example, F5 in 2019/2020 (Fig. 5) and F7 and/or F9 in 2021/2022 (Fig. 6) exhibited trajectories characterised by an initial increase, followed either by a plateau under lower storage temperatures or a decline under higher temperatures. Such patterns may be associated with tuber ageing or seed tuber quality traits, such as sprouting capacity (Zou et al. 2024). However, these factors explained only a relatively small proportion of the total variance, and the individual metabolite weakly associated with them did not display consistently clear trends. Nevertheless, identifying metabolites linked to traits associated with physiological ageing could provide valuable insights into storage-induced biological and physiological changes in potato seed tubers, thereby improving our understanding of the underlying regulatory mechanisms.

## Supplementary Information

Below is the link to the electronic supplementary material.

Supplementary file1 (DOCX 1291 KB)

## Data Availability

Data is available at 10.4121/03308b8f-dd38-4ad3-905b-bd31fa5f9880.
