# Supplementary material for: Untargeted Metabolomics Reveals Major Patterns of Metabolic Shifts in Potato Seed Tubers during Storage
Source: Potato Res. 2026 Jun 5;69(4):131. doi: 10.1007/s11540-026-10083-2 (PMC13241448; doi:10.1007/s11540-026-10083-2)
Supplement: Supplementary file 1 — Supplementary file1 (DOCX 1291 KB) [file 11540_2026_10083_MOESM1_ESM.docx]

**Untargeted metabolomics reveals major patterns of metabolic shifts in potato seed tubers during storage**

**Chunmei Zou**^1,2^, **Ric C. H. de Vos**^3^, **Roland Mumm**^3^, **Henriëtte D. L. M. van Eekelen**^3^, **Aurin M. Vos**^3^, **Alejandro Thérèse Navarro**^4^, **Robert D. Hall**^3^, **Martin K. van Ittersum**^2^, **Willemien J. M. Lommen**^1^, **Paul C. Struik**^1^

^1^Centre for Crop Systems Analysis, Wageningen University & Research, P.O. Box 430, 6700 AK Wageningen, The Netherlands

^2^Plant Production Systems, Wageningen University & Research, P.O. Box 430, 6700 AK Wageningen, The Netherlands

^3^Bioscience, Wageningen University and Research, P.O. Box 16, 6700 AA Wageningen, The Netherlands

^4^Plant Breeding, Wageningen University, P.O. Box 386, 6700 AJ Wageningen, The Netherlands. Present address: Rijk Zwaan, 4793 RS Rijnaart, The Netherlands

**Correspondence** Chunmei Zou: m.zouchunmei@gmail.com

**Supplementary Data**

Supplementary data are available at 4TU.ResearchData: <https://doi.org/10.4121/03308b8f-dd38-4ad3-905b-bd31fa5f9880>.

**Tables**

**Table S1** Number of analysed samples at each sampling moment for GC-MS and LC-MS platforms. The 12 samples at SH and S0 (pre-storage) consisted of four cultivars × three blocks. The 36 samples at S1 to S6 included four cultivars × three storage temperatures × three blocks. Other sample numbers were due to losses or poorly analysed samples

| **Storage Season** | **Platform** |  | **Sampling Timing** | | | | | | | |  | **Total** |
| --- | --- | --- | --- | --- | --- | --- | --- | --- | --- | --- | --- | --- |
|  |  |  | **SH** | **S0** | **S1** | **S2** | **S3** | **S4** | **S5** | **S6** |  |  |
| 2019/2020 | GC-MS |  | - | 12 | 36 | 36 | 36 | 36 | 36 | - |  | 192 |
|  | LC-MS |  | - | 12 | 36 | 36 | 36 | 36 | 36 | - |  | 192 |
| 2021/2022 | GC-MS |  | 12 | 10 | 35 | 35 | 34 | 36 | 35 | 33 |  | 230 |
|  | LC-MS |  | 12 | 12 | 36 | 36 | 36 | 36 | 36 | 35 |  | 239 |

**Table S2** Number of metabolites retained after each step of data filtering

| **Storage Season** | **Platform** |  | **Pre-processing** | **First Criteria** | **Second Criteria** |
| --- | --- | --- | --- | --- | --- |
| 2019/2020 | GC-MS |  | 113 | 107 | 104 |
|  | LC-MS |  | 1105 | 871 | 829 |
| 2021/2022 | GC-MS |  | 139 | 124 | 117 |
|  | LC-MS |  | 425 | 415 | 375 |

**Table S3** ANOVA result for the number of GC-MS and LC-MS metabolites in two storage seasons. Significance codes indicate ^***^ *p* < 0.001, ^**^ *p* < 0.01, ^*^ *p* < 0.05, and ‘’ ns

|  | **2019/2020** | | | | | | **2021/2022** | | | | | |
| --- | --- | --- | --- | --- | --- | --- | --- | --- | --- | --- | --- | --- |
|  | **Df** | **Sum Sq** | **Mean Sq** | **F value** | **Pr(>F)** | **Sig** | **Df** | **Sum Sq** | **Mean Sq** | **F value** | **Pr(>F)** | **Sig** |
| **GC-MS** |  |  |  |  |  |  |  |  |  |  |  |  |
| Cultivar | 3 | 297.5 | 99.15 | 27.39 | 4.9×10^-14^ | ^***^ | 3 | 936.2 | 312.07 | 66.04 | < 2×10^-16^ | ^***^ |
| Temperature | 2 | 17.6 | 8.78 | 2.43 | 0.0921 |  | 2 | 217.0 | 108.49 | 22.96 | 1.38×10^-9^ | ^***^ |
| Time | 5 | 49.4 | 9.88 | 2.73 | 0.0219 | ^*^ | 7 | 438.8 | 62.69 | 13.27 | 1.09×10^-13^ | ^***^ |
| Block | 2 | 13.9 | 6.95 | 1.92 | 0.1505 |  | 2 | 16.2 | 8.10 | 1.72 | 0.1830 |  |
| Cultivar×Temperature | 6 | 31.8 | 5.30 | 1.46 | 0.1947 |  | 6 | 28.2 | 4.70 | 0.99 | 0.4309 |  |
| Cultivar×Time | 15 | 137.1 | 9.14 | 2.53 | 0.0024 | ^**^ | 21 | 206.0 | 9.81 | 2.08 | 0.0056 | ^**^ |
| Temperature×Time | 10 | 72.3 | 7.23 | 2.00 | 0.0379 |  | 14 | 261.2 | 18.66 | 3.95 | 6.96×10^-6^ | ^***^ |
| Cultivar×Temperature×Time | 30 | 117.0 | 3.90 | 1.08 | 0.3724 |  | 42 | 239.6 | 5.71 | 1.21 | 0.2010 |  |
| Residuals | 142 | 514.1 | 3.62 |  |  |  | 176 | 831.7 | 4.73 |  |  |  |
| **LC-MS** |  |  |  |  |  |  |  |  |  |  |  |  |
| Cultivar | 3 | 236998 | 78999 | 155.13 | < 2×10^-16^ | ^***^ | 3 | 23270 | 7757 | 94.41 | < 2×10^-16^ | ^***^ |
| Temperature | 2 | 26844 | 13422 | 26.36 | 1.84×10^-10^ | ^***^ | 2 | 4270 | 2135 | 25.99 | 1.07×10^-10^ | ^***^ |
| Time | 5 | 186784 | 37357 | 73.36 | < 2×10^-16^ | ^***^ | 7 | 44610 | 6373 | 77.57 | < 2×10^-16^ | ^***^ |
| Block | 2 | 1600 | 800 | 1.57 | 0.2113 |  | 2 | 154 | 77 | 0.94 | 0.3937 |  |
| Cultivar×Temperature | 6 | 2533 | 422 | 0.83 | 0.5493 |  | 6 | 514 | 86 | 1.04 | 0.3988 |  |
| Cultivar×Time | 15 | 18545 | 1236 | 2.43 | 0.0036 | ^**^ | 21 | 4011 | 191 | 2.33 | 0.0015 | ^**^ |
| Temperature×Time | 10 | 36844 | 3684 | 7.24 | 3.54×10^-9^ | ^***^ | 14 | 8491 | 606 | 7.38 | 3.38×10^-12^ | ^***^ |
| Cultivar×Temperature×Time | 30 | 11953 | 398 | 0.78 | 0.7810 |  | 42 | 4072 | 97 | 1.18 | 0.2273 |  |
| Residuals | 142 | 72313 | 509 |  |  |  | 189 | 15527 | 82 |  |  |  |

**Table S4** ANOVA result for the number of GC-MS metabolites within cultivars in two storage seasons. Significance codes indicate ^***^ *p* < 0.001, ^**^ *p* < 0.01, ^*^ *p* < 0.05, and ‘’ ns

|  | **2019/2020** | | | | | | **2021/2022** | | | | | |
| --- | --- | --- | --- | --- | --- | --- | --- | --- | --- | --- | --- | --- |
|  | **Df** | **Sum Sq** | **Mean Sq** | **F value** | **Pr(>F)** | **Sig** | **Df** | **Sum Sq** | **Mean Sq** | **F value** | **Pr(>F)** | **Sig** |
| **Agria** |  |  |  |  |  |  |  |  |  |  |  |  |
| Temperature | 2 | 1.59 | 0.80 | 0.30 | 0.7421 |  | 2 | 51.94 | 25.97 | 6.27 | 0.0041 | ^**^ |
| Time | 5 | 86.81 | 17.36 | 6.56 | 0.0003 | ^***^ | 7 | 110.09 | 15.73 | 3.80 | 0.0027 | ^**^ |
| Block | 2 | 8.04 | 4.02 | 1.52 | 0.2335 |  | 2 | 4.86 | 2.43 | 0.59 | 0.5609 |  |
| Temperature×Time | 10 | 23.30 | 2.33 | 0.88 | 0.5600 |  | 14 | 75.19 | 5.37 | 1.30 | 0.2490 |  |
| Residuals | 34 | 89.96 | 2.65 |  |  |  | 43 | 178.12 | 4.14 |  |  |  |
| **Festien** |  |  |  |  |  |  |  |  |  |  |  |  |
| Temperature | 2 | 15.26 | 7.63 | 1.07 | 0.3560 |  | 2 | 80.94 | 40.47 | 10.24 | 0.0003 | ^***^ |
| Time | 5 | 63.26 | 12.65 | 1.77 | 0.1460 |  | 7 | 159.81 | 22.83 | 5.77 | 0.0001 | ^***^ |
| Block | 2 | 32.70 | 16.35 | 2.29 | 0.1170 |  | 2 | 3.17 | 1.59 | 0.40 | 0.6720 |  |
| Temperature×Time | 10 | 86.07 | 8.61 | 1.20 | 0.3240 |  | 14 | 232.17 | 16.58 | 4.19 | 0.0002 | ^***^ |
| Residuals | 34 | 243.30 | 7.16 |  |  |  | 40 | 158.16 | 3.95 |  |  |  |
| **Innovator** |  |  |  |  |  |  |  |  |  |  |  |  |
| Temperature | 2 | 0.93 | 0.46 | 0.27 | 0.7677 |  | 2 | 17.58 | 8.79 | 3.64 | 0.0342 | ^*^ |
| Time | 5 | 17.48 | 3.50 | 2.01 | 0.1018 |  | 7 | 100.99 | 14.43 | 5.98 | 5.91×10^-5^ | ^***^ |
| Block | 2 | 9.59 | 4.80 | 2.76 | 0.0775 |  | 2 | 1.97 | 0.98 | 0.41 | 0.6679 |  |
| Temperature×Time | 10 | 32.85 | 3.29 | 1.89 | 0.0815 |  | 14 | 35.36 | 2.53 | 1.05 | 0.4283 |  |
| Residuals | 34 | 59.07 | 1.74 |  |  |  | 45 | 108.59 | 2.41 |  |  |  |
| **Lady Claire** |  |  |  |  |  |  |  |  |  |  |  |  |
| Temperature | 2 | 31.59 | 15.80 | 6.35 | 0.0046 | ^**^ | 2 | 99.90 | 49.93 | 5.49 | 0.0076 | ^**^ |
| Time | 5 | 18.98 | 3.80 | 1.53 | 0.2080 |  | 7 | 270.00 | 38.57 | 4.24 | 0.0013 | ^**^ |
| Block | 2 | 0.70 | 0.35 | 0.14 | 0.8687 |  | 2 | 8.30 | 4.15 | 0.46 | 0.6365 |  |
| Temperature×Time | 10 | 47.07 | 4.71 | 1.89 | 0.0814 |  | 14 | 159.70 | 11.41 | 1.26 | 0.2752 |  |
| Residuals | 34 | 84.63 | 2.49 |  |  |  | 42 | 381.90 | 9.10 |  |  |  |

**Table S5** ANOVA result for the number of LC-MS metabolites within cultivars in two storage seasons. Significance codes indicate ^***^ *p* < 0.001, ^**^ *p* < 0.01, ^*^ *p* < 0.05, and ‘’ ns

|  | **2019/2020** | | | | | | **2021/2022** | | | | | |
| --- | --- | --- | --- | --- | --- | --- | --- | --- | --- | --- | --- | --- |
|  | **Df** | **Sum Sq** | **Mean Sq** | **F value** | **Pr(>F)** | **Sig** | **Df** | **Sum Sq** | **Mean Sq** | **F value** | **Pr(>F)** | **Sig** |
| **Agria** |  |  |  |  |  |  |  |  |  |  |  |  |
| Temperature | 2 | 5416 | 2708 | 4.93 | 0.0132 | ^*^ | 2 | 2613 | 1307 | 15.18 | 9.17×10^-6^ | ^***^ |
| Time | 5 | 45229 | 9046 | 16.46 | 3.01×10^-8^ | ^***^ | 7 | 10829 | 1547 | 17.97 | 3.91×10^-11^ | ^***^ |
| Block | 2 | 192 | 96 | 0.17 | 0.8408 |  | 2 | 222 | 111 | 1.29 | 0.2860 |  |
| Temperature×Time | 10 | 12167 | 1217 | 2.21 | 0.0413 | ^*^ | 14 | 4216 | 301 | 3.50 | 0.0007 | ^***^ |
| Residuals | 34 | 18686 | 550 |  |  |  | 45 | 3875 | 86 |  |  |  |
| **Festien** |  |  |  |  |  |  |  |  |  |  |  |  |
| Temperature | 2 | 3958 | 1979 | 5.51 | 0.0085 | ^**^ | 2 | 1067 | 534 | 10.94 | 0.0001 | ^***^ |
| Time | 5 | 20476 | 4095 | 11.40 | 1.64×10^-6^ | ^***^ | 7 | 7647 | 1093 | 22.40 | 7.55×10^-13^ | ^***^ |
| Block | 2 | 47 | 23 | 0.07 | 0.94 |  | 2 | 146 | 73 | 1.49 | 0.2357 |  |
| Temperature×Time | 10 | 5045 | 504 | 1.41 | 0.22 |  | 14 | 2794 | 200 | 4.09 | 0.0001 | ^***^ |
| Residuals | 34 | 12211 | 359 |  |  |  | 46 | 2244 | 49 |  |  |  |
| **Innovator** |  |  |  |  |  |  |  |  |  |  |  |  |
| Temperature | 2 | 9680 | 4840 | 10.73 | 0.0002 | ^***^ | 2 | 553 | 277 | 2.66 | 0.0806 |  |
| Time | 5 | 82621 | 16524 | 36.62 | 9.47×10^-13^ | ^***^ | 7 | 9582 | 1369 | 13.17 | 3.61×10^-9^ | ^***^ |
| Block | 2 | 56 | 28 | 0.06 | 0.94 |  | 2 | 349 | 174 | 1.68 | 0.1979 |  |
| Temperature×Time | 10 | 16084 | 1608 | 3.56 | 0.0026 | ^**^ | 14 | 3192 | 228 | 2.19 | 0.0231 | ^*^ |
| Residuals | 34 | 15344 | 451 |  |  |  | 46 | 4780 | 104 |  |  |  |
| **Lady Claire** |  |  |  |  |  |  |  |  |  |  |  |  |
| Temperature | 2 | 10323 | 5162 | 7.95 | 0.0015 | ^**^ | 2 | 661 | 330 | 3.97 | 0.0257 | ^*^ |
| Time | 5 | 57003 | 11401 | 17.56 | 1.41×10^-8^ | ^***^ | 7 | 20452 | 2922 | 35.09 | < 2×10^-16^ | ^***^ |
| Block | 2 | 5299 | 2649 | 4.08 | 0.0258 | ^*^ | 2 | 239 | 119 | 1.43 | 0.2488 |  |
| Temperature×Time | 10 | 15501 | 1550 | 2.39 | 0.0287 | ^*^ | 14 | 2359 | 169 | 2.02 | 0.0370 | ^*^ |
| Residuals | 34 | 22079 | 649 |  |  |  | 46 | 3830 | 83 |  |  |  |

**Table S6** Factor-explained (% of total) variance for the top 10 factors by cultivar-specific FA and the corresponding number of metabolites with absolute loading > 0.3 (italicised)

| **Cultivar** | **Total Factor Number** |  | **F1** | **F2** | **F3** | **F4** | **F5** | **F6** | **F7** | **F8** | **F9** | **F10** |
| --- | --- | --- | --- | --- | --- | --- | --- | --- | --- | --- | --- | --- |
| **2019/2020** |  |  |  |  |  |  |  |  |  |  |  |  |
| Agria | 33 |  | 25.0% | 10.8% | 8.7% | 3.5% | 2.4% | 1.5% | 1.4% | 1.0% | 1.0% | 0.8% |
|  |  |  | *469* | *278* | *118* | *76* | *34* | *9* | *16* | *6* | *17* | *11* |
| Festien | 36 |  | 28.1% | 6.8% | 6.1% | 4.5% | 3.8% | 3.4% | 1.2% | 0.9% | 0.8% | 0.7% |
|  |  |  | *516* | *127* | *109* | *52* | *70* | *80* | *15* | *8* | *9* | *7* |
| Innovator | 31 |  | 32.5% | 7.1% | 5.5% | 4.4% | 3.4% | 1.7% | 1.0% | 1.0% | 0.9% | 0.9% |
|  |  |  | *516* | *157* | *75* | *100* | *60* | *23* | *9* | *8* | *6* | *11* |
| Lady Claire | 32 |  | 29.7% | 11.1% | 5.1% | 2.6% | 1.9% | 1.8% | 1.7% | 1.6% | 1.2% | 0.8% |
|  |  |  | *574* | *278* | *47* | *42* | *34* | *34* | *24* | *27* | *12* | *8* |
| **2021/2022** |  |  |  |  |  |  |  |  |  |  |  |  |
| Agria | 17 |  | 27.5% | 19.5% | 14.1% | 9.5% | 4.4% | 1.2% | 1.2% | 0.8% | 0.7% | 0.6% |
|  |  |  | *242* | *126* | *109* | *116* | *51* | *5* | *8* | *1* | *1* | *2* |
| Festien | 17 |  | 30.2% | 16.8% | 14.7% | 9.9% | 3.5% | 1.5% | 0.7% | 0.7% | 0.7% | 0.5% |
|  |  |  | *262* | *117* | *133* | *107* | *25* | *10* | *6* | *5* | *1* | *2* |
| Innovator | 19 |  | 37.3% | 15.9% | 7.6% | 5.0% | 3.6% | 2.6% | 1.9% | 1.8% | 1.7% | 1.0% |
|  |  |  | *294* | *112* | *75* | *54* | *46* | *22* | *11* | *6* | *7* | *4* |
| Lady Claire | 16 |  | 29.2% | 13.5% | 12.6% | 11.8% | 8.8% | 1.7% | 0.9% | 0.6% | 0.6% | 0.5% |
|  |  |  | *232* | *80* | *107* | *127* | *87* | *5* | *4* | *0* | *2* | *1* |

**Figures**


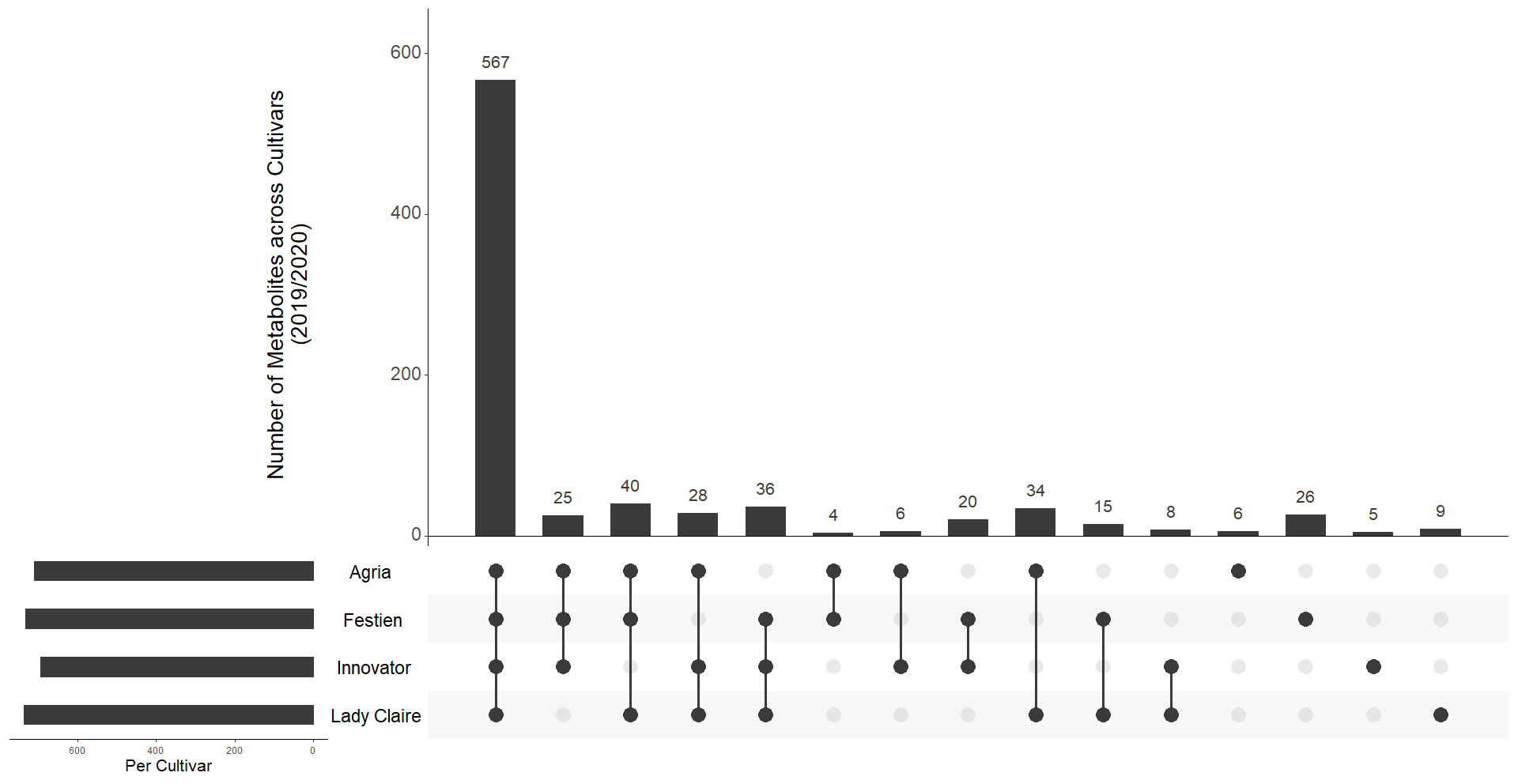


**Fig. S1** Number of LC-MS metabolites in the 2019/2020 storage season across four cultivars


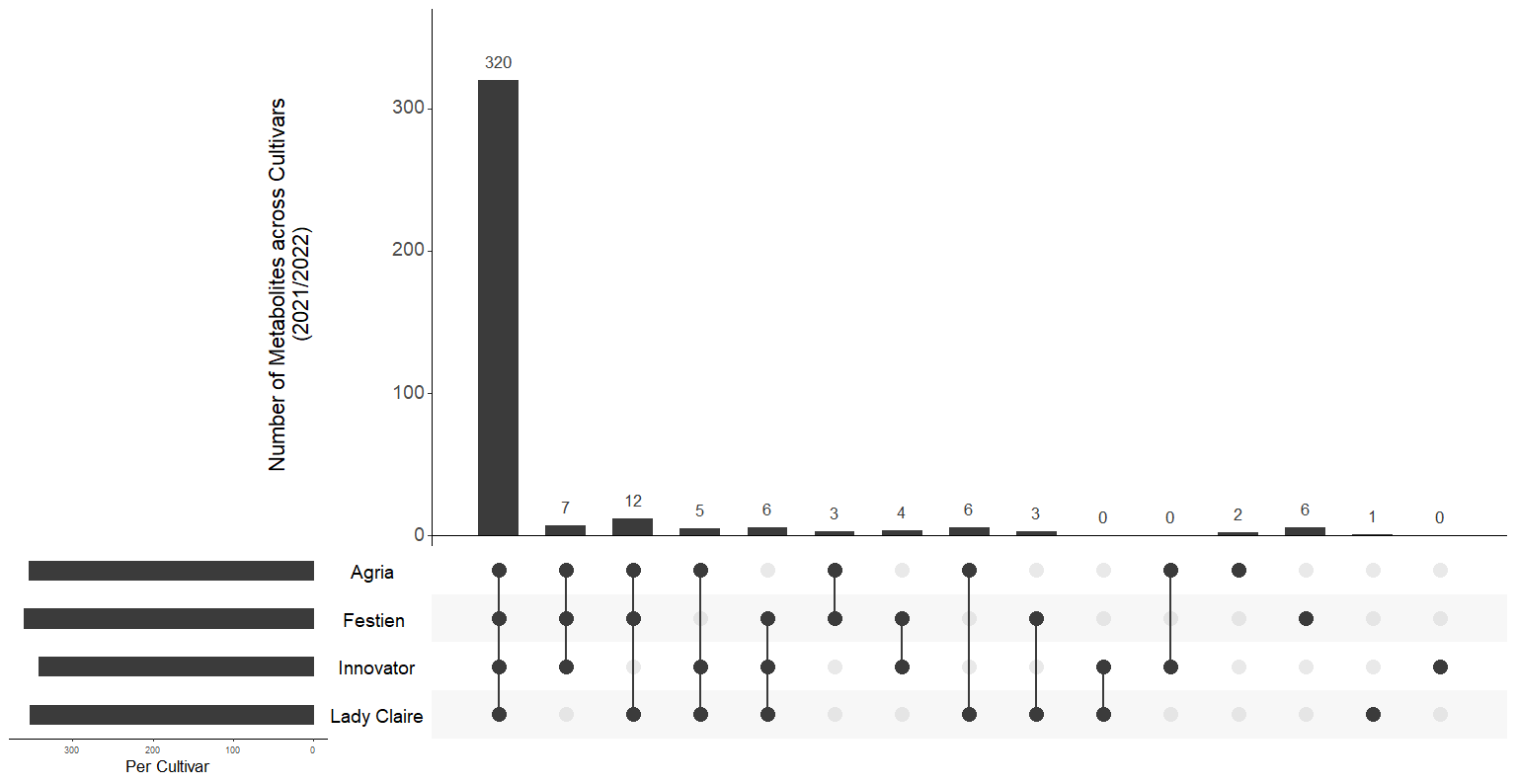


**Fig. S2** Number of LC-MS metabolites in the 2021/2022 storage season across four cultivars


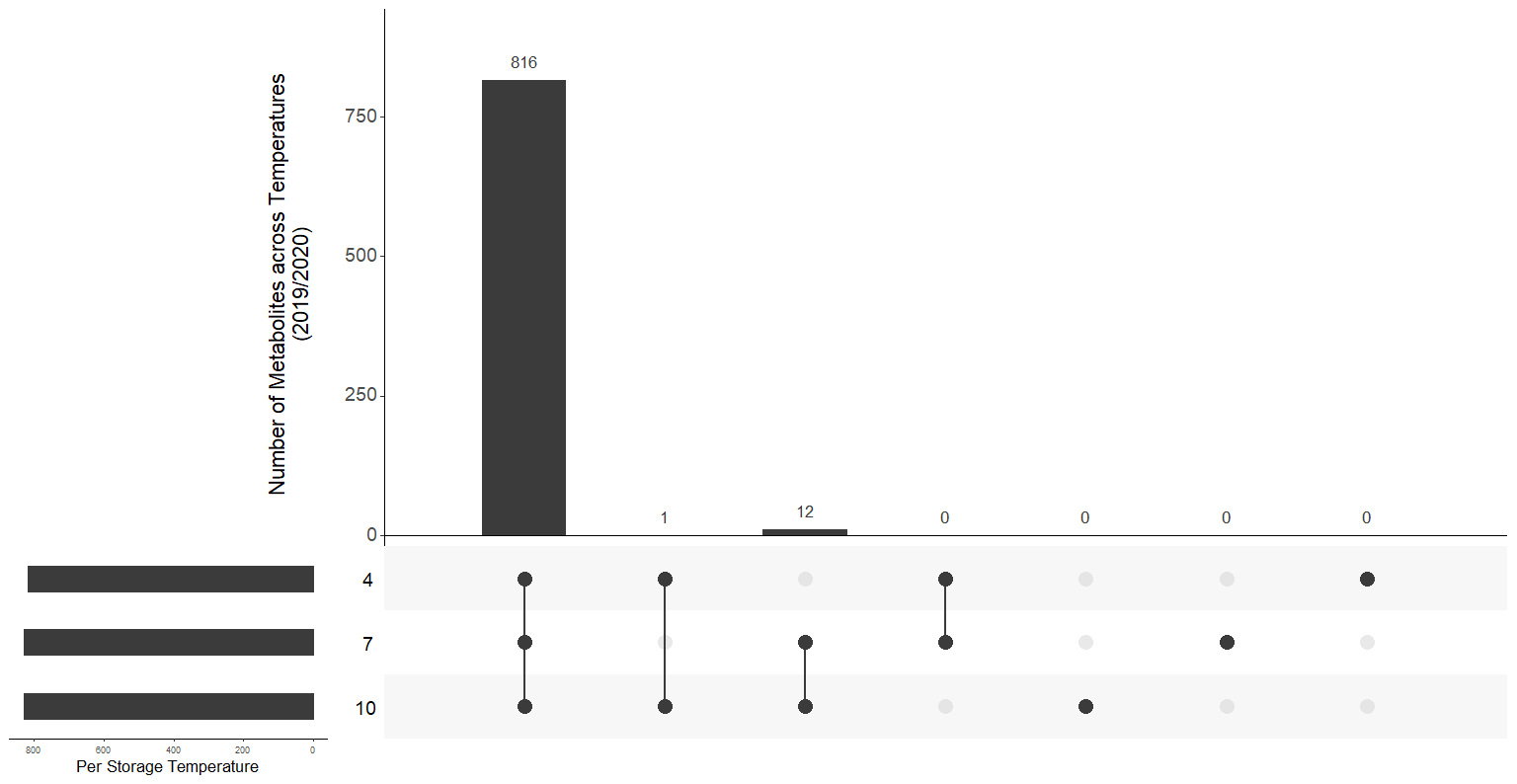


**Fig. S3** Number of LC-MS metabolites in the 2019/2020 storage season across three storage temperatures


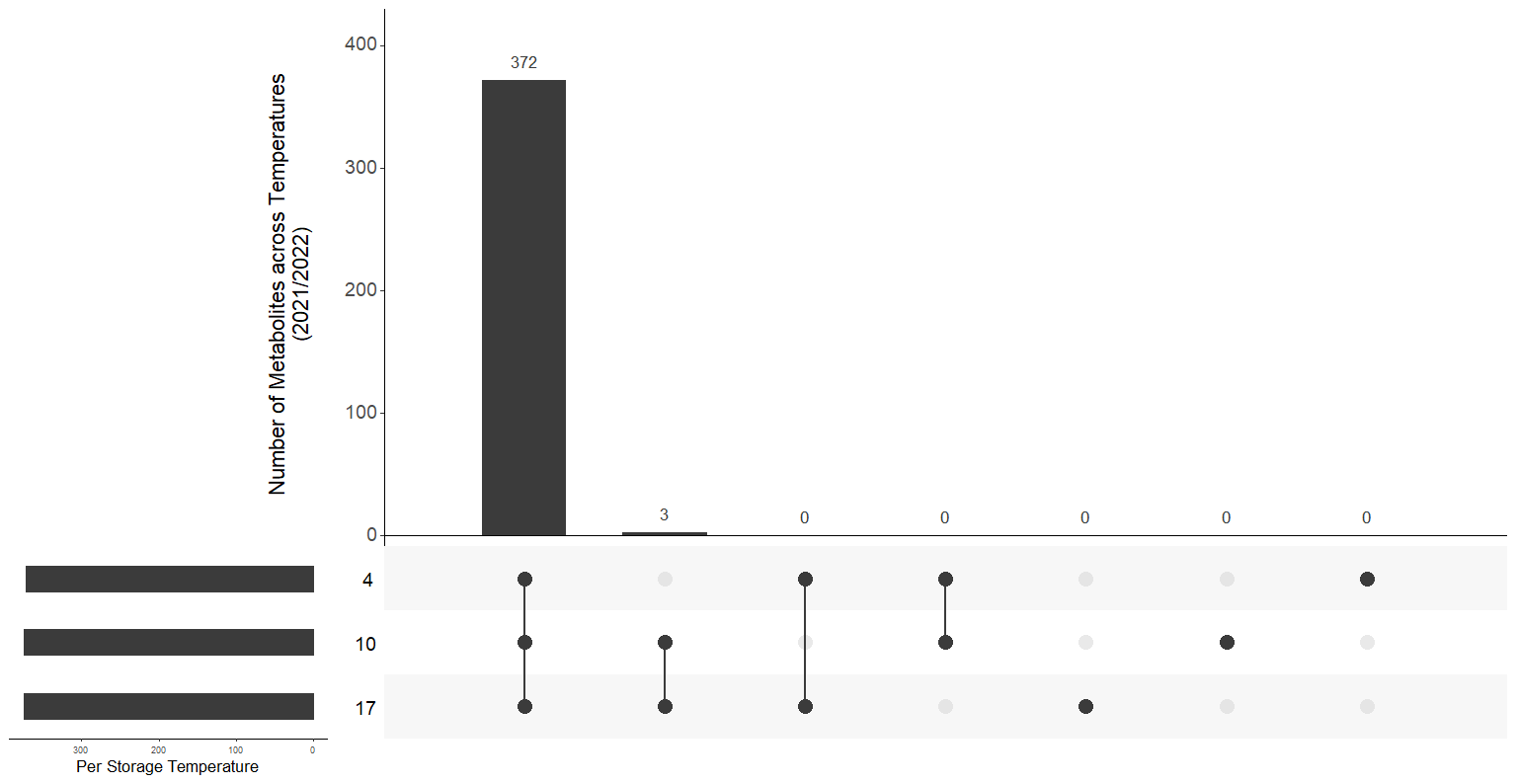


**Fig. S4** Number of LC-MS metabolites in the 2021/2022 storage season across three storage temperatures


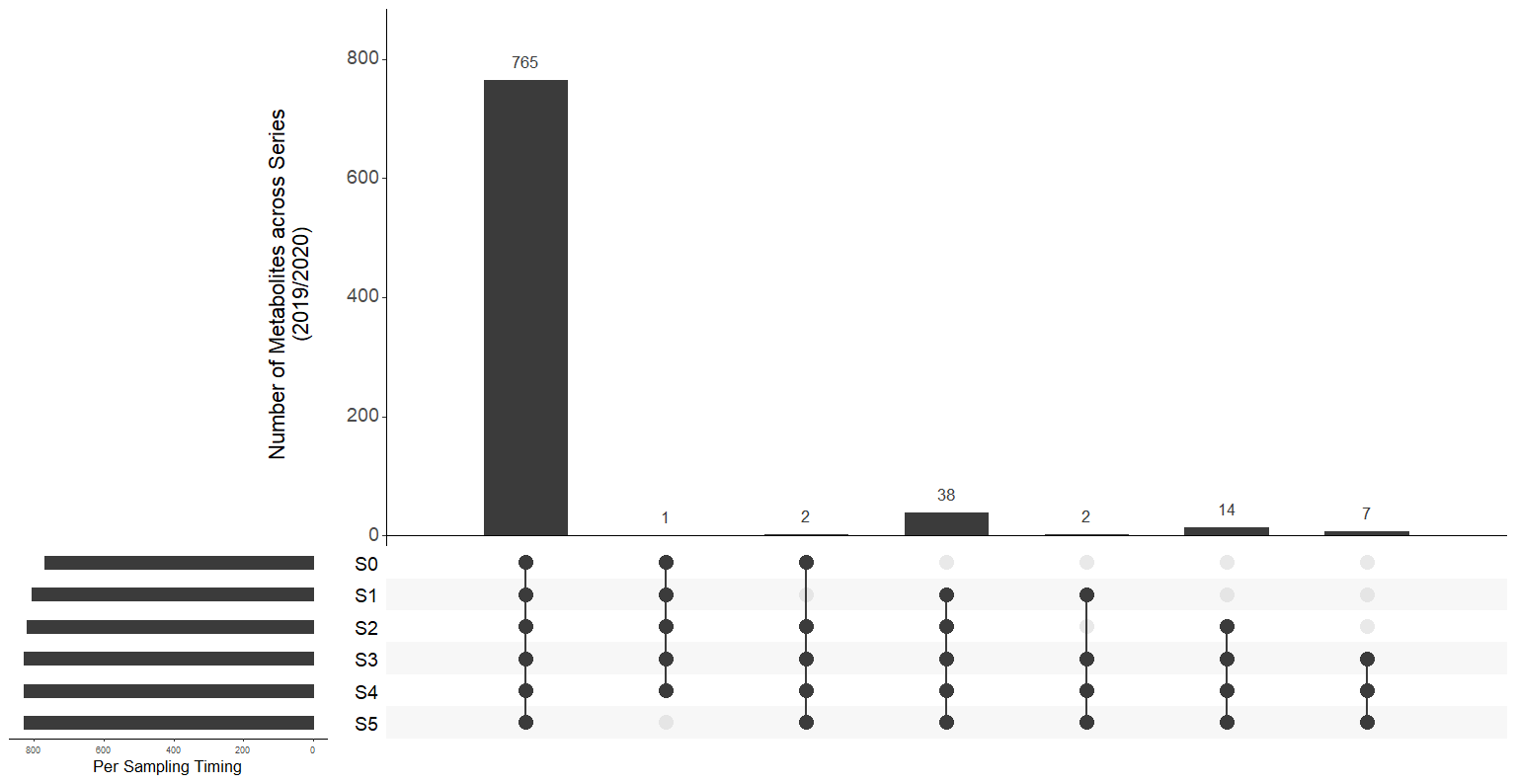


**Fig. S5** Number of LC-MS metabolites in the 2019/2020 storage season across six series. Zero intersections are not shown due to graphic limitation


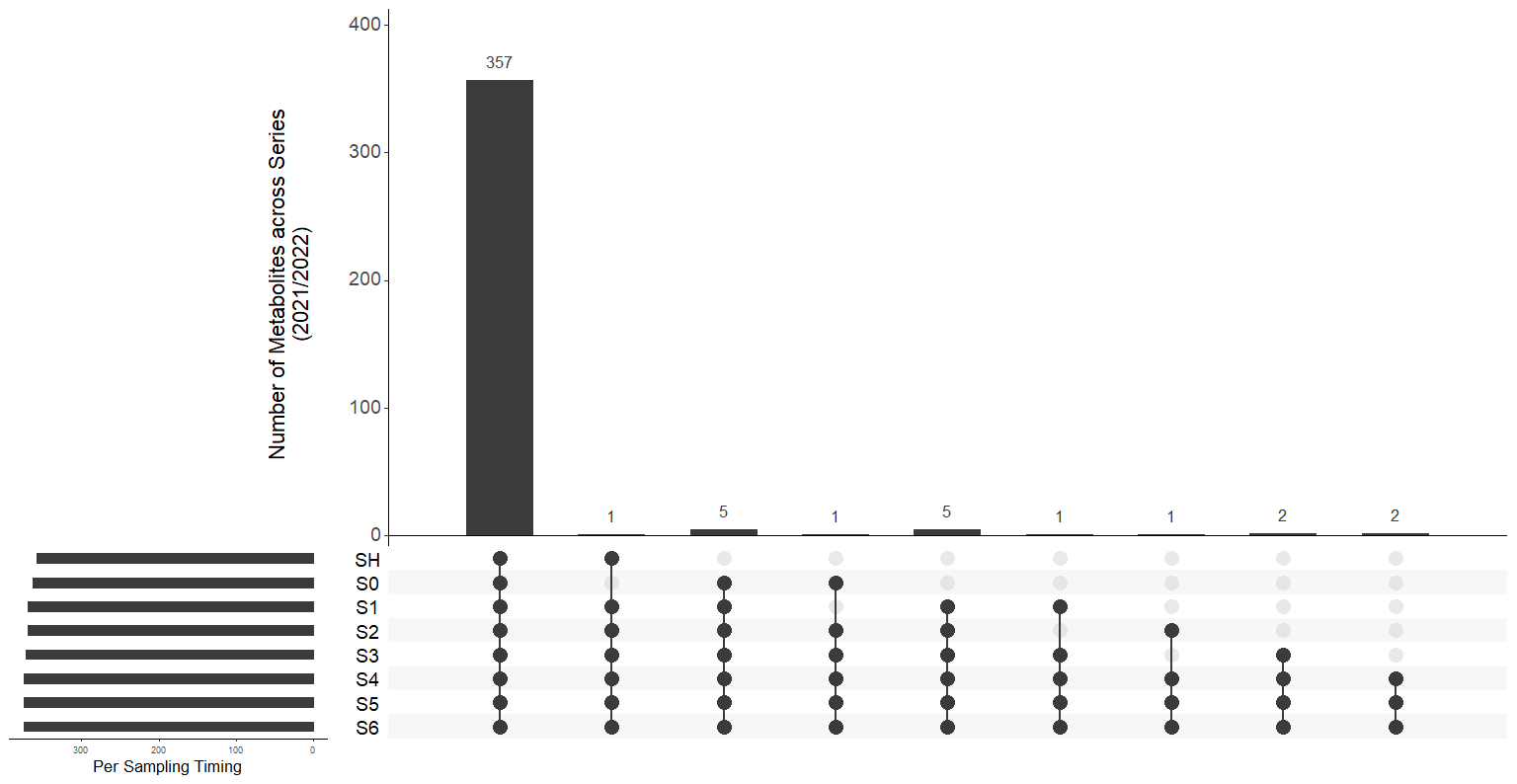


**Fig. S6** Number of LC-MS metabolites in the 2021/2022 storage season across eight series. Zero intersections are not shown due to graphic limitation


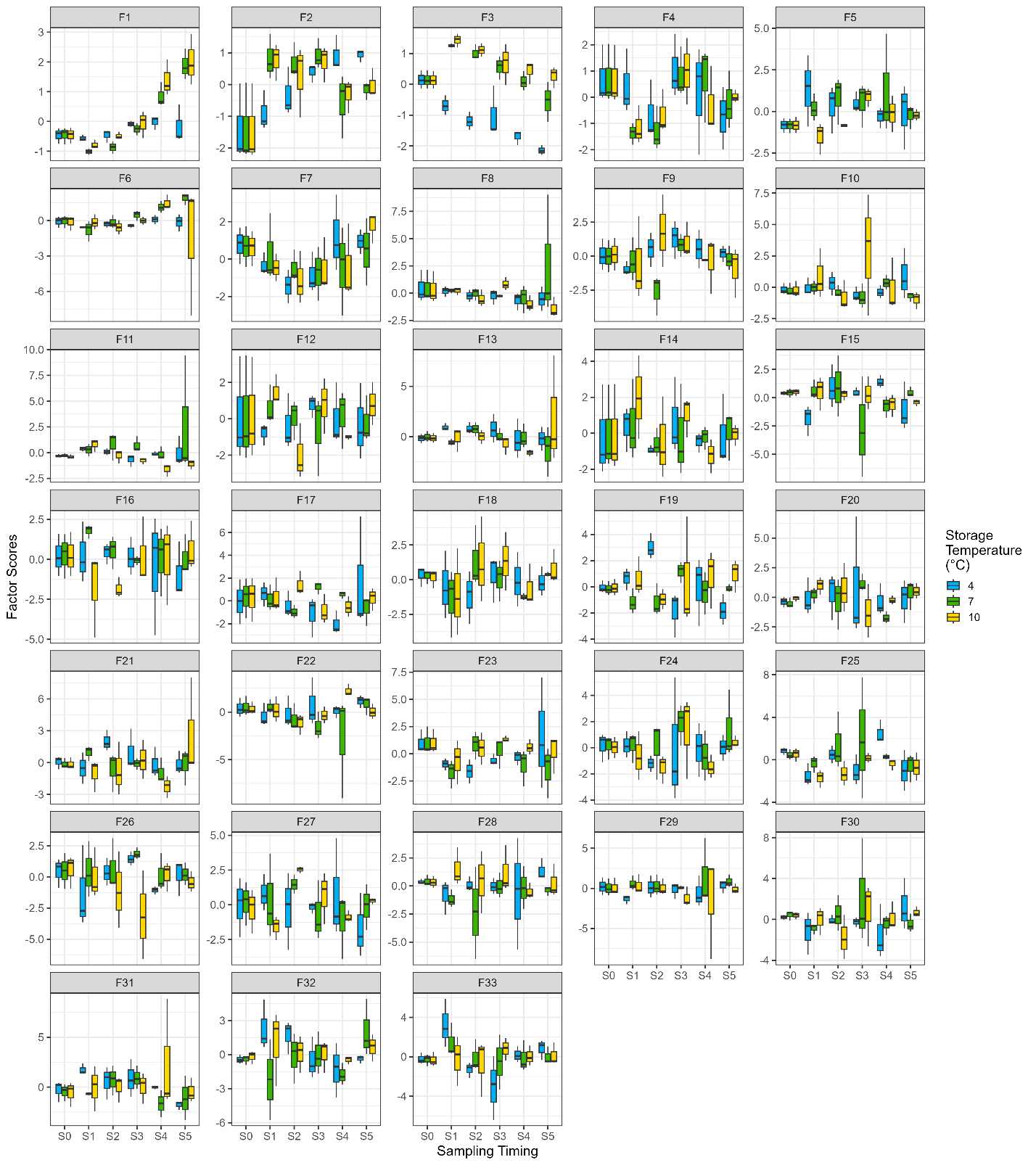


**Fig. S7** Factor scores of tuber samples of Agria stored at three temperatures (represented by colours) and sampled across six time points in the 2019/2020 storage season


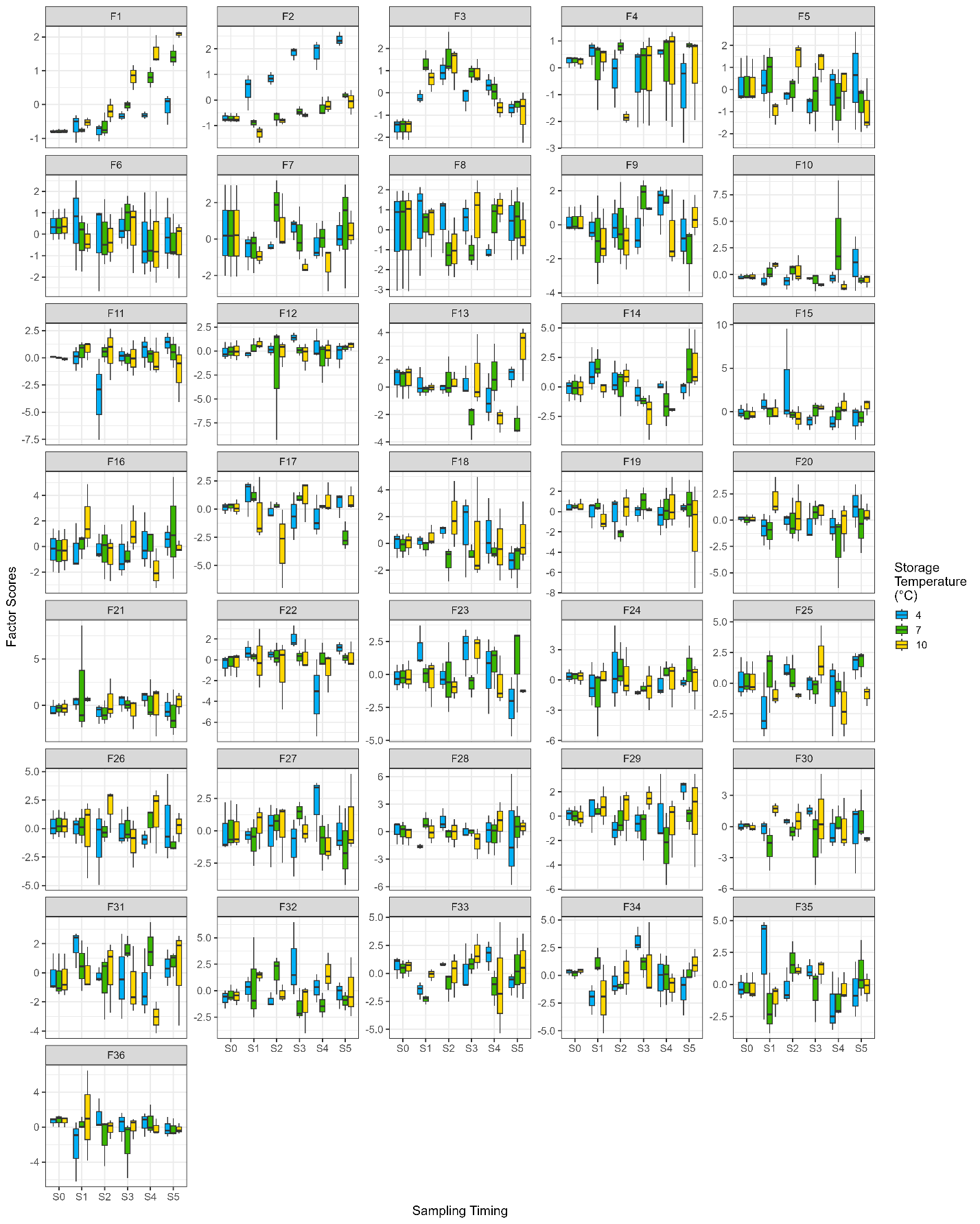


**Fig. S8** Factor scores of tuber samples of Festien stored at three temperatures (represented by colours) and sampled across six time points in the 2019/2020 storage season


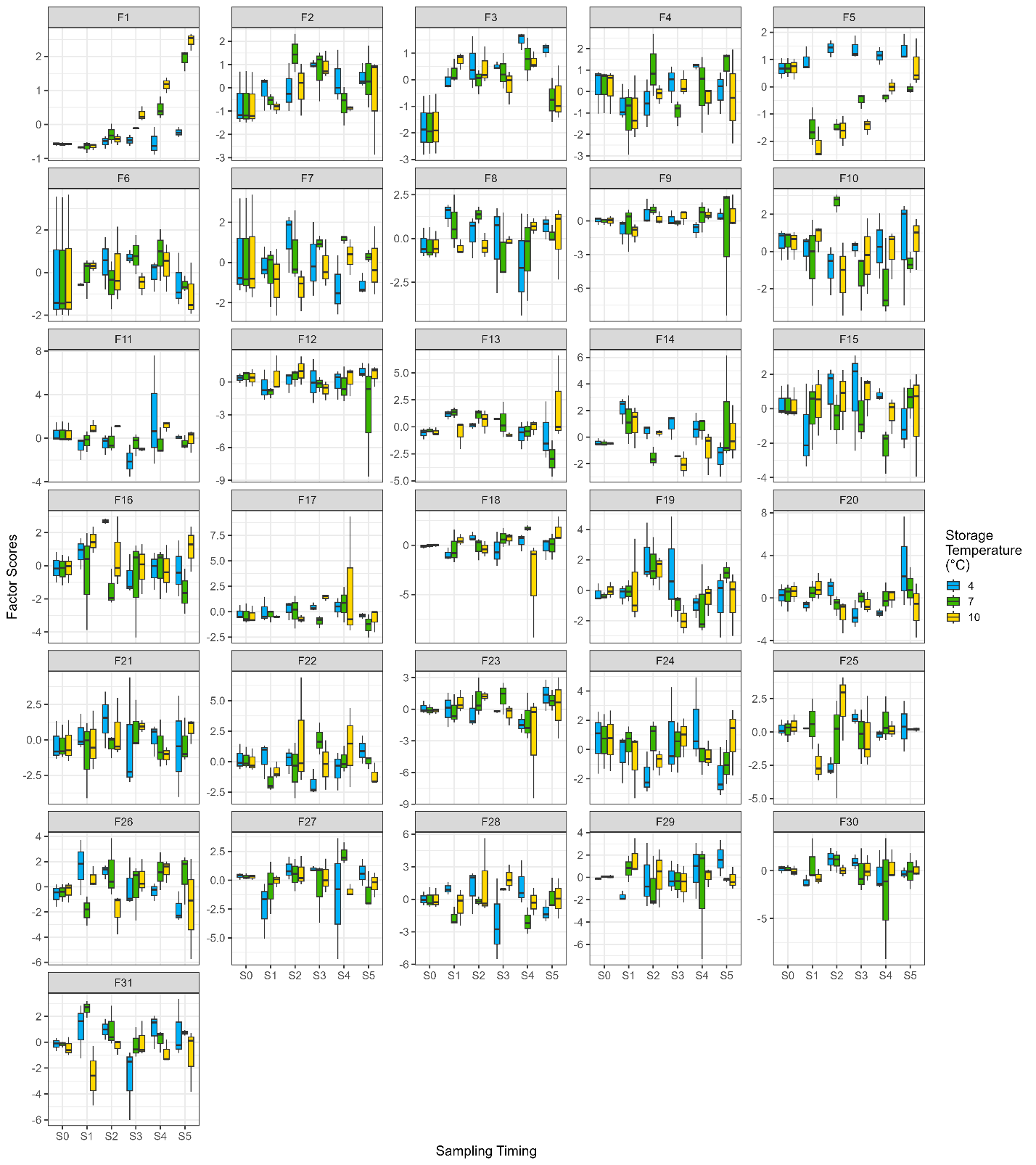


**Fig. S9** Factor scores of tuber samples of Innovator stored at three temperatures (represented by colours) and sampled across six time points in the 2019/2020 storage season


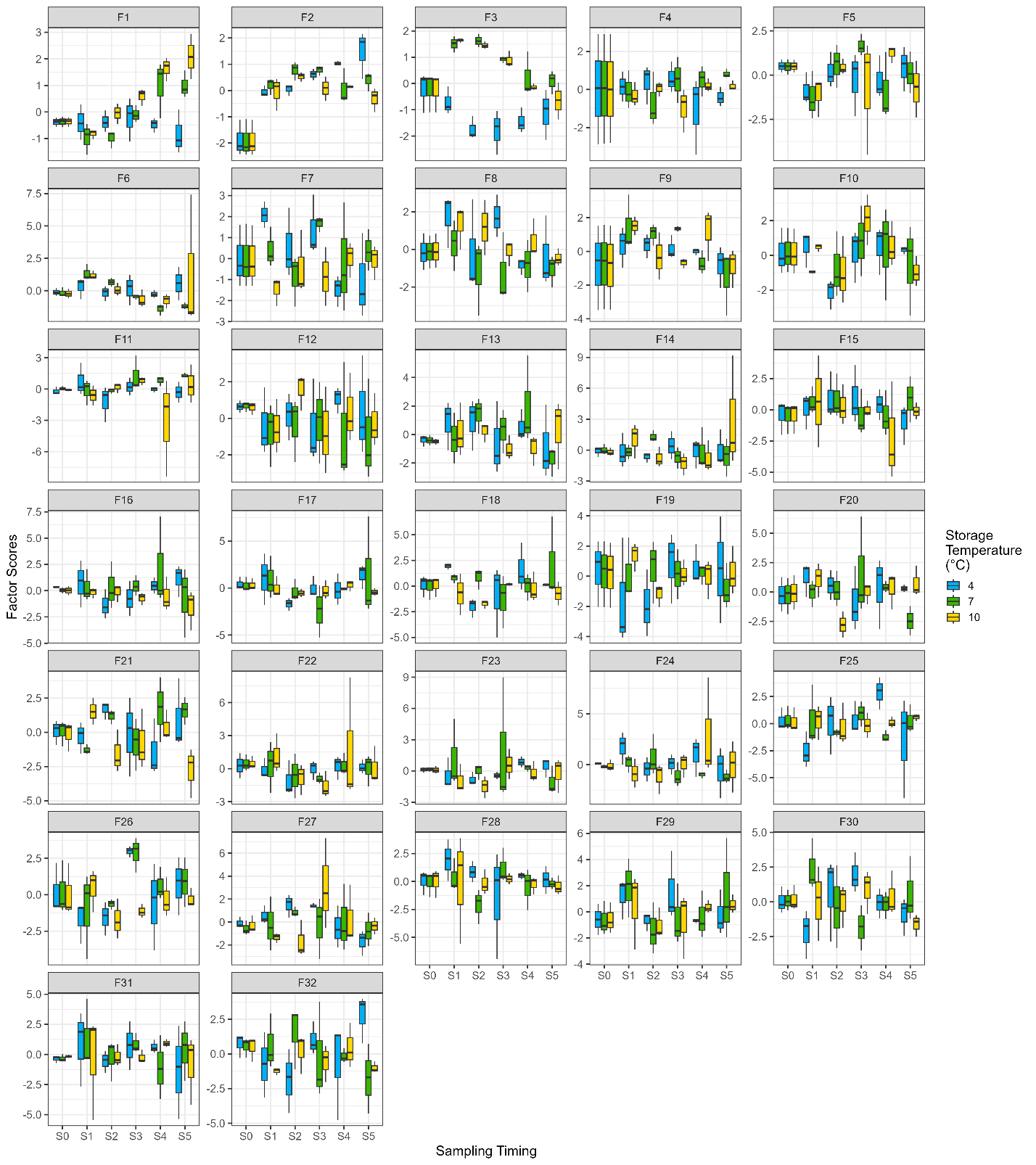


**Fig. S10** Factor scores of tuber samples of Lady Claire stored at three temperatures (represented by colours) and sampled across six time points in the 2019/2020 storage season


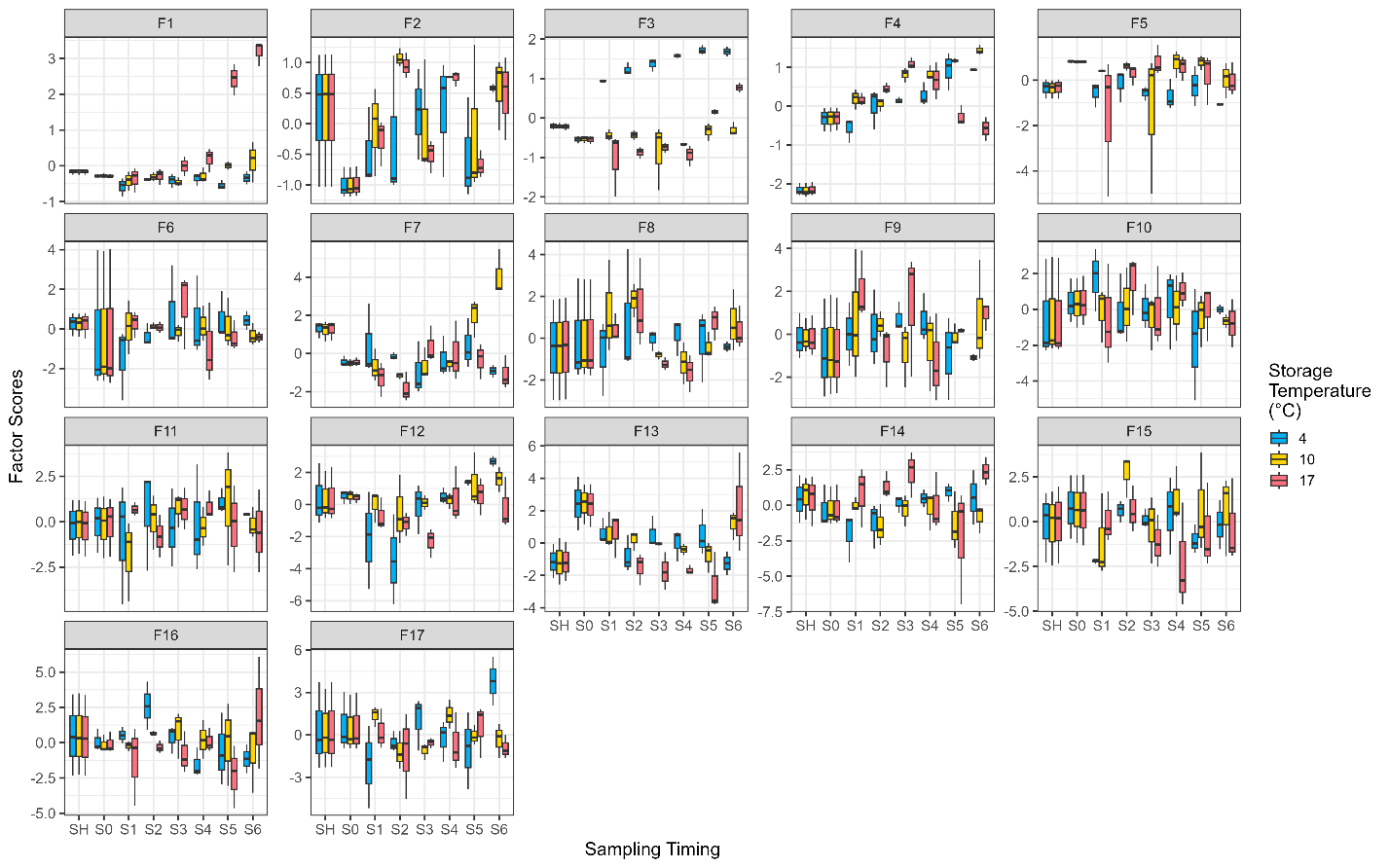


**Fig. S11** Factor scores of tuber samples of Agria in the 2021/2022 storage season stored at three temperatures (represented by colours) and sampled across eight time points


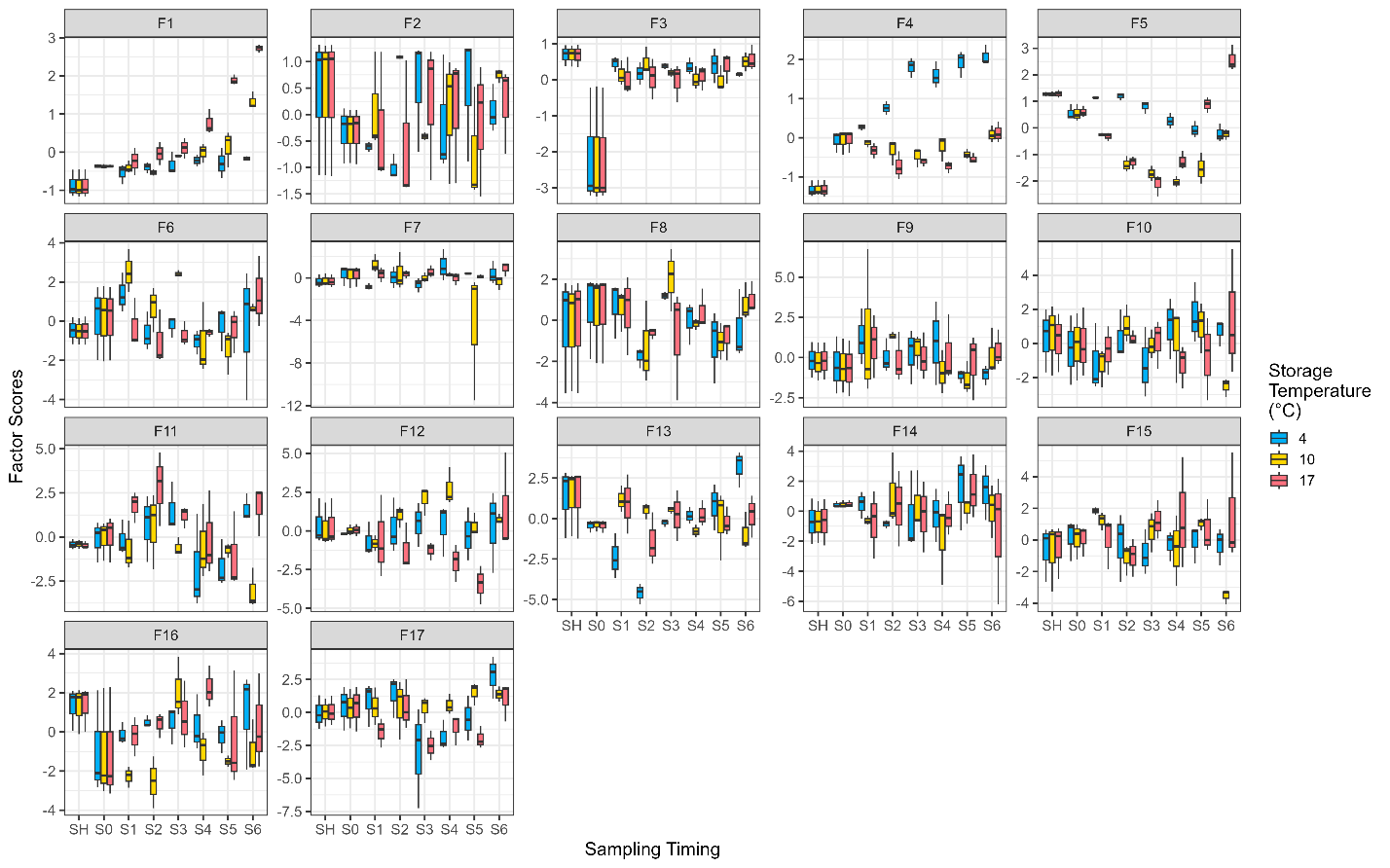


**Fig. S12** Factor scores of tuber samples of Festien in the 2021/2022 storage season stored at three temperatures (represented by colours) and sampled across eight time points


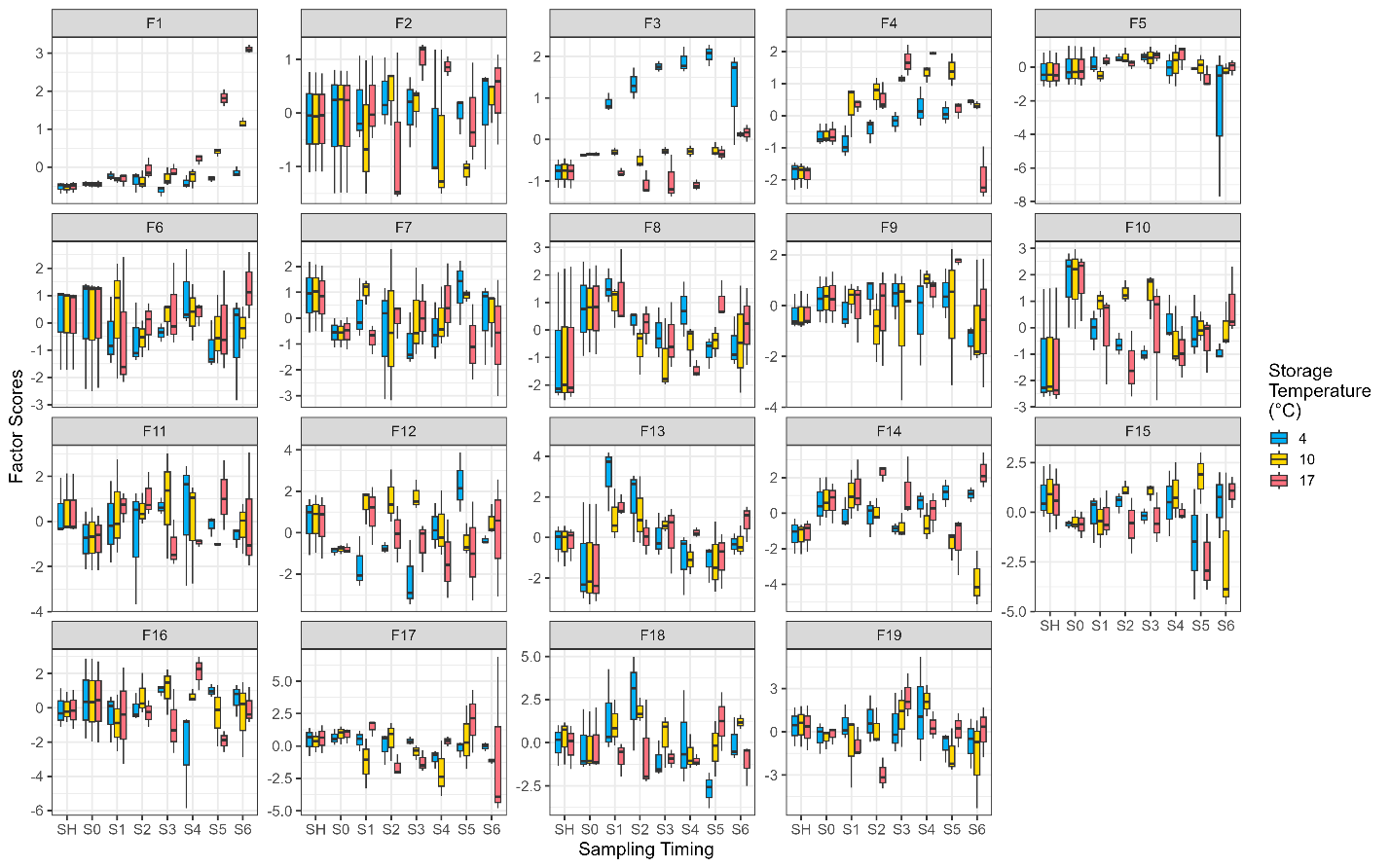


**Fig. S13** Factor scores of tuber samples of Innovator in the 2021/2022 storage season stored at three temperatures (represented by colours) and sampled across eight time points


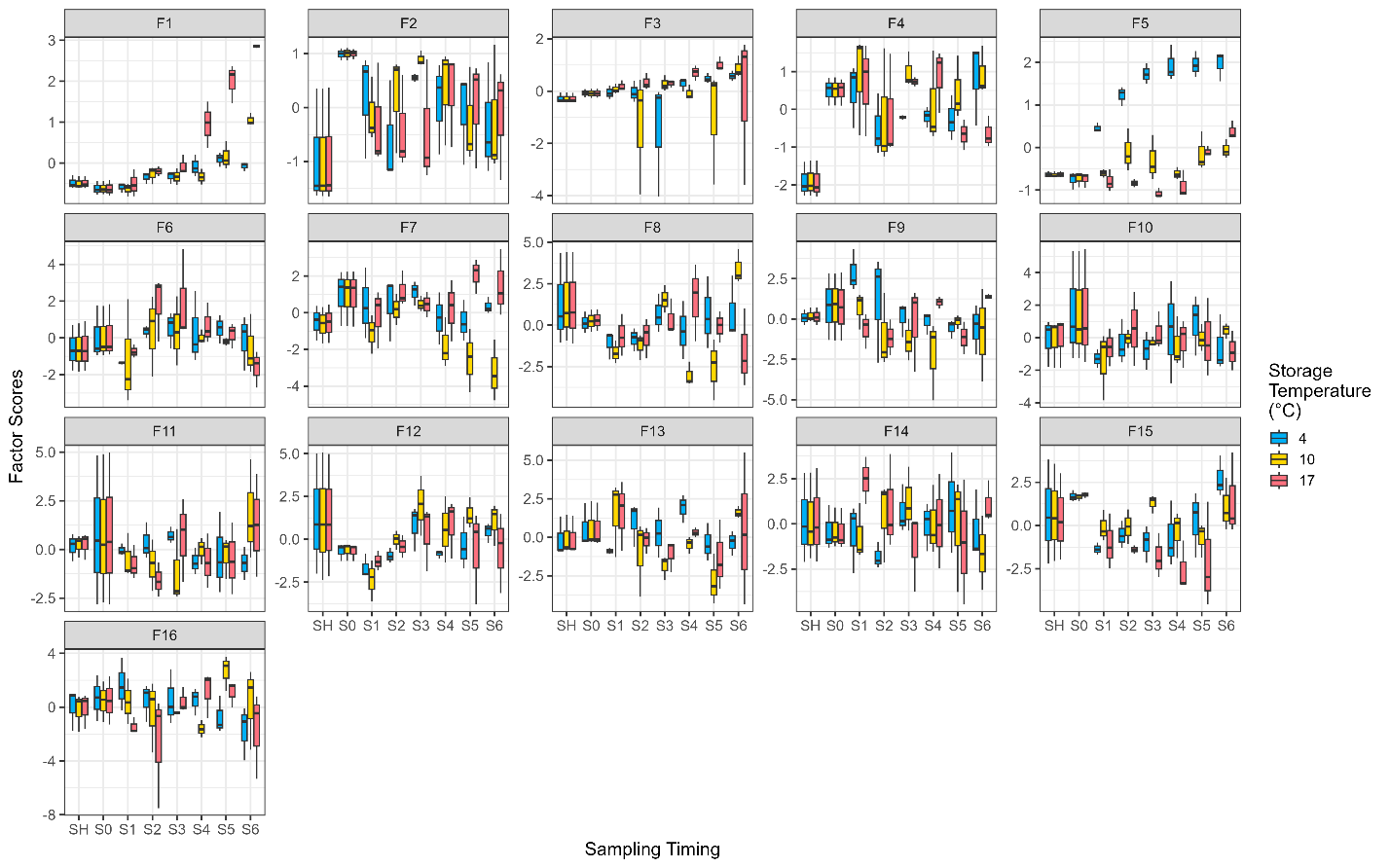
**Fig. S14** Factor scores of tubers samples of cultivar Lady Claire in the 2021/2022 storage season stored at three temperatures (represented by colours) and sampled across eight time points
